# CDC7 inhibition impairs neuroendocrine transformation in lung and prostate tumors through MYC degradation

**DOI:** 10.1038/s41392-024-01908-y

**Published:** 2024-07-26

**Authors:** Alvaro Quintanal-Villalonga, Kenta Kawasaki, Esther Redin, Fathema Uddin, Swanand Rakhade, Vidushi Durani, Amin Sabet, Moniquetta Shafer, Wouter R. Karthaus, Samir Zaidi, Yingqian A. Zhan, Parvathy Manoj, Harsha Sridhar, Dennis Kinyua, Hong Zhong, Barbara P. Mello, Metamia Ciampricotti, Umesh K. Bhanot, Irina Linkov, Juan Qiu, Radhika A. Patel, Colm Morrissey, Sanjoy Mehta, Jesse Barnes, Michael C. Haffner, Nicholas D. Socci, Richard P. Koche, Elisa de Stanchina, Sonia Molina-Pinelo, Sohrab Salehi, Helena A. Yu, Joseph M. Chan, Charles M. Rudin

**Affiliations:** 1 Department of Medicine, Thoracic Oncology Service, Memorial Sloan Kettering Cancer Center, New York, NY USA; 2 Cancer Biology and Genetics Program, Sloan Kettering Institute, Memorial Sloan Kettering Cancer Center, New York, NY USA; 3 Vagelos College of Physicians and Surgeons, Columbia University, New York, NY USA; 4 Weill Cornell Graduate School of Medical Sciences, Weill Cornell Medicine, New York, NY USA; 5 Human Oncology and Pathogenesis Program, Memorial Sloan Kettering Cancer Center, New York, NY USA; 6 Center for Epigenetics Research, Memorial Sloan Kettering Cancer Center, New York, NY USA; 7 Pathology Core Facility, Department of Pathology and Laboratory Medicine, MSKCC, New York, NY USA; 8 Antitumor Assessment Core, Memorial Sloan Kettering Cancer Center, New York, NY USA; 9 Divisions of Human Biology and Clinical Research, Fred Hutchinson Cancer Center, Seattle, WA USA; 10 Department of Urology, University of Washington, Seattle, WA USA; 11 Gene Editing & Screening Core Facility, Memorial Sloan Kettering Cancer Center, New York, NY USA; 12 Department of Laboratory Medicine and Pathology, University of Washington, Seattle, WA USA; 13 Bioinformatics Core Facility, Memorial Sloan Kettering Cancer Center, New York, NY USA; 14 Institute of Biomedicine of Seville (IBiS), HUVR, CSIC, Universidad de Sevilla, Seville, Spain; 15 Computational Oncology, Department of Epidemiology and Biostatistics, Memorial Sloan Kettering Cancer Center, New York, NY USA; 16 Weill Cornell Medical College, New York, NY USA; 17 Present Address: Swiss Institute for Experimental Cancer Research (ISREC), School of Life Sciences, EPFL, Lausanne, Switzerland

**Keywords:** Drug development, Lung cancer, Urological cancer

## Abstract

Neuroendocrine (NE) transformation is a mechanism of resistance to targeted therapy in lung and prostate adenocarcinomas leading to poor prognosis. Up to date, even if patients at high risk of transformation can be identified by the occurrence of Tumor Protein P53 (*TP53)* and Retinoblastoma Transcriptional Corepressor 1 *(RB1)* mutations in their tumors, no therapeutic strategies are available to prevent or delay histological transformation. Upregulation of the cell cycle kinase Cell Division Cycle 7 (CDC7) occurred in tumors during the initial steps of NE transformation, already after *TP53/RB1* co-inactivation, leading to induced sensitivity to the CDC7 inhibitor simurosertib. CDC7 inhibition suppressed NE transdifferentiation and extended response to targeted therapy in in vivo models of NE transformation by inducing the proteasome-mediated degradation of the MYC Proto-Oncogen (MYC), implicated in stemness and histological transformation. Ectopic overexpression of a degradation-resistant MYC isoform reestablished the NE transformation phenotype observed on targeted therapy, even in the presence of simurosertib. CDC7 inhibition also markedly extended response to standard cytotoxics (cisplatin, irinotecan) in lung and prostate small cell carcinoma models. These results nominate CDC7 inhibition as a therapeutic strategy to constrain lineage plasticity, as well as to effectively treat NE tumors de novo or after transformation. As simurosertib clinical efficacy trials are ongoing, this concept could be readily translated for patients at risk of transformation.

## Introduction

Lineage plasticity mediates histologic transformation, a phenomenon most extensively described in the context of resistance to targeted therapies.^1–3^ Transformation to aggressive, neuroendocrine (NE) derivatives resembling small cell lung carcinoma (SCLC) occurs in up to 14% of metastatic Epidermal Growth Factor Receptor (*EGFR)-*mutant lung adenocarcinomas (LUAD) treated with EGFR tyrosine kinase inhibitors,^4^ and in over 20% of metastatic androgen receptor (AR)-dependent prostate adenocarcinomas (PRADs) treated with enzalutamide.^5^ NE-transformed tumors are typically treatment-refractory and rapidly progressive, associated with a poor prognosis similar to or worse than that of de novo SCLC.^5–7^

Concurrent inactivation of two key tumor suppressor genes, Tumor Protein P53 (TP53) and Retinoblastoma Transcriptional Corepressor 1 (RB1), is a common hallmark of NE transformation^6,8^ and among adenocarcinomas defines the population at highest risk of transformation.^6^ Extensive epigenomic reprogramming and multiple additional molecular alterations have been implicated as contributors to NE transformation,^2,9^ including the Phosphoinositide 3-kinase (PI3K)/Protein Kinase B (AKT) pathway,^1,10,11^ Fibroblast growth factor receptor (FGFR)/Janus Kinase (JAK)/ Signal transducers and activators of transcription (STAT) signaling,^12^ and key transcriptional regulators such as the SRY-Box Transcription Factor 2 (SOX2) and the MYC Proto-Oncogene (MYC).^1,3^ Despite progress in defining the biology of NE transformation and in characterizing patients with adenocarcinoma at increased risk of NE transformation,^6^ to date no therapies are available to effectively constrain plasticity and suppress transformation. The identification of druggable targets to prevent transformation is an unmet clinical need.

We performed a dependency screen in a transformed SCLC (T-SCLC) preclinical model and identified Cell Division Cycle 7 (CDC7) as a therapeutic vulnerability in NE tumors. Due to its known roles in the initiation of DNA replication and DNA damage response,^13–15^ CDC7 has been considered a promising therapeutic target in cancer, for which a number of inhibitors are under investigation. These include simurosertib, which has shown single agent and combinatorial activity with cytotoxics in preclinical models of multiple cancer types.^16^ A phase I clinical study of simurosertib has defined safety, tolerability, and a recommended dose for subsequent studies,^17^ and phase II single agent efficacy evaluation is ongoing (ClinicalTrials.gov; NCT03261947).

We provide evidence for a role of CDC7 in NE transformation in both lung and prostate cancers, where a dependency on CDC7 is induced upon *TP53* and *RB1* inactivation, before full histologic transformation. CDC7 inhibition with simurosertib suppresses targeted therapy-induced NE transformation in vivo and prolongs adenocarcinoma response to therapy. We show this effect is attributable to induction of proteasomal degradation of MYC, which is essential for transformation. Simurosertib also extends response to cytotoxic chemotherapy in de novo SCLC and in lung and prostate-transformed NE patient-derived xenograft (PDX) models. Our data nominate CDC7 as a therapeutic target in the lung and prostate settings, both to prevent NE transformation in tumors at high risk, and to improve therapeutic response in de novo or post-transformation high-grade NE tumors.

## Results

### CDC7 is upregulated early in NE transformation

**Fig. 1:**
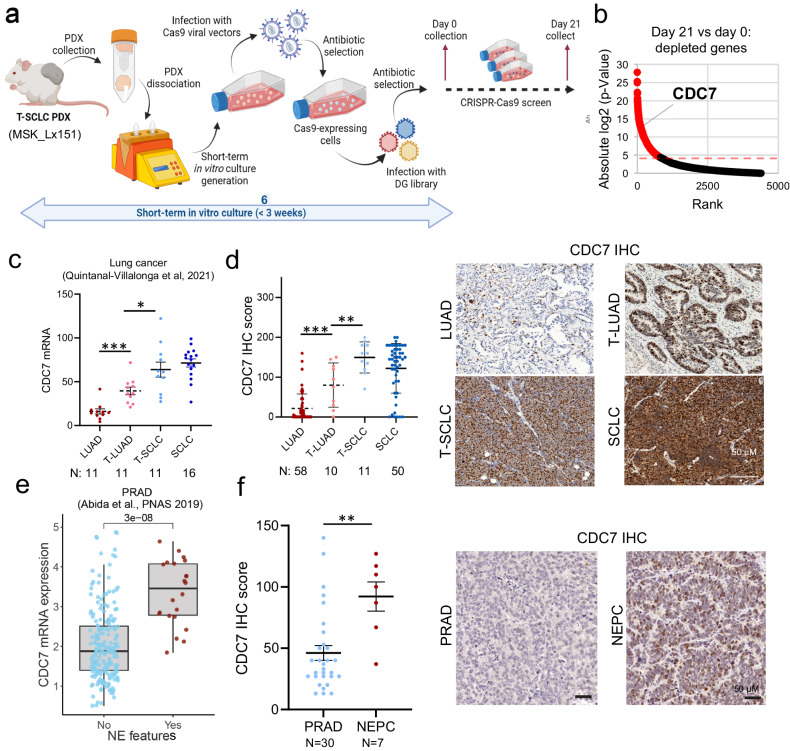
CDC7 is upregulated during NE transformation in lung and prostate tumors. **a** Schematic of the generation of a PDX-derived, short-term cultured cell line generation, derived from the T-SCLC PDX Lx_151, and handling for the performance of CRISPR-Cas9 KO screen to identify therapeutic vulnerabilities of T-SCLC. Created with BioRender.com. **b** Plot showing genes for which sgRNAs were depleted in the CRISPR-Cas9 KO screen, ranked by p-value. Red dots are indicative of genes passing the p-value threshold. CDC7 mRNA expression **c** and protein abundance (**d**) in lung cancer clinical specimens, categorized as control never transformed adenocarcinomas (LUAD, RNA *n* = 11, protein *n* = 46), transforming adenocarcinomas (T-LUAD, RNA *n* = 11, protein *n* = 10) and small cell carcinomas (T-SCLC, RNA *n* = 11, protein *n* = 20) and control de novo small cell carcinomas (SCLC, RNA *n* = 16, protein *n* = 50). For (**d**), H-score medians and standard deviations (right) and representative immunohistochemistry (IHC) images (left) are shown. (**e**) CDC7 mRNA expression in prostate adenocarcinoma (PRAD) tumors with (*n* = 22) or without (*n* = 210) NE features (data from.^22^ (**f**) CDC7 protein expression in PRAD (*n* = 30) and NEPC (*n* = 7) PDX, as assessed by IHC. H-score medians and standard deviation (right) and representative images (left) are shown. p-value legend: *<0.05, **<0.01, ***<0.001

To identify potential therapeutic vulnerabilities of tumors undergoing NE transformation, we performed a Clustered Regularly Interspaced Short Palindromic Repeats (CRISPR)-Cas9 drop-out screen over 15 population doublings (21 days) of a short-term in vitro cultured cell line derived from a *EGFR*-mutant transformed SCLC (T-SCLC) PDX (SCLC-N subtype), previously described in ref. ^18^ (Fig. 1a). We leveraged a previously reported druggable genome single guide (sg) RNA library with ~14,000 sgRNAs targeting ~2300 genes encoding targets of known pharmacological inhibitors in clinical development or The Food and Drugs Administration (FDA)-approved drugs.^19^ Deep sequencing analyses comparing sgRNA abundance of final timepoint versus day 0 identified multiple sgRNAs targeting *CDC7* among the top hits depleted in the screen (Fig. 1b and Supplementary Data S1), thus nominating this gene as a potential dependency in T-SCLC. CDC7 is a cell cycle regulator with serine/threonine kinase activity. Binding to its co-factor DBF4-CDC7 Kinase Regulatory Subunit (DBF4) during the late G1–to–S phase by binding to its co-factor, CDC7 phosphorylates the minichromosome maintenance 2 (MCM2) proteins to initiate DNA synthesis.^20^ The CDC7 kinase also has been implicated in maintenance of DNA replication forks and DNA damage response pathways, particularly under conditions of replication stress.^13,20^

The involvement of CDC7 in DNA replication and DNA damage response was consonant with a potential dependency of SCLC on CDC7, as this cancer type is characterized by an exceptional highly proliferative index and universal inactivation of key tumor cell cycle checkpoint factors (i.e. *TP53* and *RB1*) which might increase dependence on remaining intact regulators of cell cycle progression. This observation, together with the availability of potent and clinically safe CDC7 inhibitors, currently under clinical testing,^17^ motivated us to pursue this target. Assessment of *CDC7* mRNA expression across the spectrum of tumors represented in the Cancer Cell Line Encyclopedia (CCLE)^21^ suggested that *CDC7* expression was higher in SCLC than in any other tumor type (Supplementary Fig. s1a). Additionally, generation of isogenic SCLC cell lines with CDC7 knock out (KO) (Supplementary Fig. s1b) or overexpression (Supplementary Fig. s1c) indicated that CDC7 expression was associated with increased proliferation (Supplementary Fig. s1d, e) and colony formation in soft agar assays (Supplementary Fig s1f, g). These results highlighted a potential dependency of SCLC on CDC7, that prompted us to further explore CDC7 as a therapeutic target in NE transformation.

We observed increased *CDC7* mRNA expression in LUAD that subsequently gave rise to NE transformation (“transforming” LUAD, or T-LUAD) vs those that never transformed, and further upregulation in post-transformation SCLC tumors (T-SCLC) and de novo SCLC (Fig. 1c). Immunohistochemical (IHC) staining of CDC7 in clinical specimens and PDX confirmed these results at the protein level (Fig. 1d). Mimicking the results observed in lung cancer, analysis of a publicly available transcriptomic dataset of human PRAD^22^ revealed increased *CDC7* mRNA expression on those PRAD exhibiting NE features (Fig. 1e), and consistently, NEPC PDX exhibited higher CDC7 protein expression than those derived from PRAD (Fig. 1f).

### *TP53/RB1* inactivation induces CDC7 expression and dependency

**Fig. 2:**
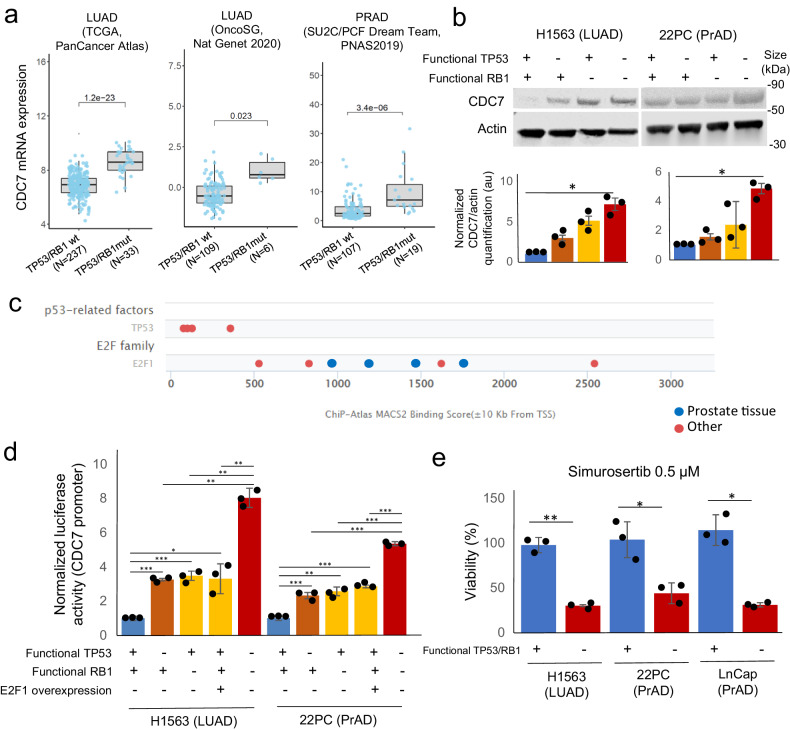
TP53 and RB1 inactivation drive CDC7 upregulation in lung and prostate adenocarcinoma. **a** CDC7 mRNA expression in adenocarcinoma clinical specimens, categorized by TP53/RB1 mutational status. Data obtained from LUAD TCGA (PanCancer, *n* = 237 wild type (wt), 33 mutated), LUAD OncoSG (*n* = 109 wt, 6 mutated)^45^ and PRAD SU2C/PCF Dream Team (*n* = 107 wt, 19 mutated)^46^ (**b**) Western blot images showing CDC7 protein expression in isogenic H1563 (LUAD) and 22PC (PRAD) cell lines with or without induced loss of function of TP53 and/or RB1 by shRNA against RB1 and dominant negative TP53 gene overexpression (H1563) or CRISPR/Cas9 knock out (22PC). Representative western blot image is shown (top) with quantification shown, as volume of the CDC7 band normalized by the volume of actin, and again normalized to control expression, which takes the value of 1. **c** Binding score for TP53 and E2F1 in the transcription start site of the CDC7 gene in different experimental settings including specimens from prostate and other tissues. Data obtained from The Signaling Pathways Project (ChIP-seq Atlas). **d** Barplot exhibiting data from CDC7 gene promoter reporter assays in isogenic H1563 (LUAD) and 22PC (PRAD) cell lines with or without induced inactivation of TP53 and/or and RB1, or with E2F1 overexpression. Normalized luciferase activity of a representative biological replicate is shown. **e** Barplot showing a representative biological replicate of an experiment assessing viability of control and TP53/RB1-inactivated H1563 (LUAD), 22PC and LnCap/AR (PRAD) cells treated with 0.5 µM simurosertib. Each condition shown was normalized to their matched untreated condition and represented as a normalized percentage for viability. For **d** and **e**, *p*-values were calculated using the Student’s *t* test (unpaired, heterogeneous variances, two-tailed). *p*-value legend: *<0.05, **<0.01, ***<0.001, ns, not significant

Our data indicated that CDC7 upregulation occurs early in the transformation process, being already evident in T-LUAD (Fig. 1c, d). Inactivation of both *TP53* and *RB1*, occurring either by genomic alterations or protein downregulation,^2,6,8^ is an early biomarker of high risk of NE transformation.^6,8^ Interestingly, inactivation of either of these tumor suppressor genes separately has been associated with increased *CDC7* expression in tumor types including LUAD.^15,23^ Analysis of publicly available transcriptomic datasets of clinical adenocarcinoma specimens showed increased expression of *CDC7* mRNA in LUAD and PRAD with concurrent *TP53* and *RB1* mutations relative to double wild type (wt) tumors (Fig. 2a). Consistent with previous reports,^15^ the data available showed increased *CDC7* expression in LUAD harboring *TP53* mutations but retaining wt *RB1*, as compared to double wild *TP53/RB1*-wild type tumors (Supplementary Fig. s2a), suggesting that *TP53* disfunction alone may be able to induce *CDC7* expression in this setting (Supplementary Fig. s2a). Interestingly, this was not observed in the PRAD cohort (Supplementary Fig. s2a), where only samples with *RB1* mutation, independently of the *TP53* status, showed increased CDC7 expression to that observed in the double wild type tumors (Supplementary Fig. s2a). However, the assessment of the potential contribution of loss of function of each of the genes individually was challenging due to the low number of samples showing *RB1* genomic alterations without co-occurring *TP53* mutations (Supplementary Fig. s2a). Nonetheless, *CDC7* upregulation was observed consistently in double *TP53/RB1*-mutant adenocarcinomas in all cohorts under study (Fig. 2a). Remarkably, analysis for the whole The Cancer Genome Atlas (TCGA) PanCancer dataset (*n* = 10,071) confirmed that *TP53*- or *RB1*-inactivated tumors exhibit higher CDC7 levels than *TP53/RB1*-wild type tumors, and that double *TP53/RB1*-inactivated tumors exhibit even higher CDC7 expression (Supplementary Fig. s2b), suggestive of a tumor-agnostic, universal interplay between these tumor suppressors and CDC7 dependency.

We assessed CDC7 protein levels in *TP53-* and/or *RB1*-inactivated LUAD (H1563) and PRAD (22PC) isogenic cell lines by western blot (Fig. 2b and Supplementary Fig. s2c). In H1563, loss of function of either gene induced CDC7 expression compared to control (Fig. 2b), while in the 22PC PRAD cell line, no significant increase of CDC7 was observed after individual loss of either gene. However, consistent with the clinical data (Supplementary Fig. s2a), double *TP53/RB1* inactivation induced CDC7 expression in both cell lines (Fig. 2b).

Assay for transposase-accessible chromatin (ATAC)-seq data on these cell lines^18^ did not reveal increased CDC7 gene promoter accessibility after inactivation of *TP53/RB1* (Supplementary Fig. s2d), suggesting an alternative mechanism of CDC7 upregulation. Leveraging publicly available chromatin immunoprecipitation (ChIP)-seq datasets,^24^ we observed that TP53 is reported to bind proximal to the transcriptional start site of the *CDC7* gene in a number of datasets (Fig. 2c). Similarly, the E2F Transcription Factor 1 (E2F1), a primary transcription factor activated upon *RB1* inactivation, showed high affinity binding to the *CDC7* gene transcriptional start site. Such observations suggested that TP53 and E2F1 might directly regulate *CDC7* transcription by binding to the *CDC7* gene promoter. To further explore this hypothesis, we leveraged promoter reporter assays for the *CDC7* gene locus in isogenic cell lines derived from H1563 and 22PC (Fig. 2d). In both cell lines, *TP53* inactivation led to increased *CDC7* promoter activity, suggesting that TP53 binding could repress *CDC7* gene expression. Similarly, inactivation of *RB1* led to increased CDC7 promoter activity, at levels comparable to those achieved by E2F1 overexpression (Fig. 2d), suggesting that induction of CDC7 gene expression following *RB1* inactivation might be mediated by E2F1. When concurrently inactivated, *TP53* and *RB1* increased CDC7 promoter activity to the highest levels reported in our assay (Fig. 2d). Additionally, we treated these two cell lines with palbociclib, an inhibitor of CDK4/6 able to induce dephosphorylarion (and thus activation) of Rb (Supplementary Fig. s2e). Palbociclib-reduced Rb phosphorylation levels correlated with decreased CDC7 promoter reporter activity (Supplementary Fig. s2f). These data suggest that TP53 might repress CDC7 expression by directly binding the CDC7 promoter, whereas *RB1* inactivation-driven CDC7 upregulation might occur via E2F1 binding.

Treatment of matched isogenic cell lines H1563, 22PC, and an additional PRAD line (LnCap/AR), confirmed increased sensitivity to the CDC7 inhibitor simurosertib in *TP53/RB1-*inactivated cells relative controls (Fig. 2e) or single gene loss-of-function counterparts (Supplementary Fig. s2g), even if simurosertib activity was confirmed equally in control and *TP53/RB1-*inactivated cells, as per the observed abrogation of MCM2 phosphorylation (Supplementary Fig. s2h). Consistent with prior results reporting the high selectivity of simurosertib for CDC7,^14^ we observed significant correlations of simurosertib in vitro sensitivity with CDC7 mRNA (*p* = −0.612, *p* = 0.035) and protein (*p* = −0.806, *p* = 0.002) levels (Supplementary Fig. s3a), as well as loss of simurosertib sensitivity upon CDC7 KO in SCLC cell lines Supplementary Fig. 3b and c). Taken together, these results suggest that loss of *TP53* and *RB1* activity in LUAD and PRAD, early events in NE transformation, induces dependency on CDC7, nominating it as a therapeutic target to prevent NE transformation.

### CDC7 inhibition impedes transformation and extends targeted therapy response

**Fig. 3:**
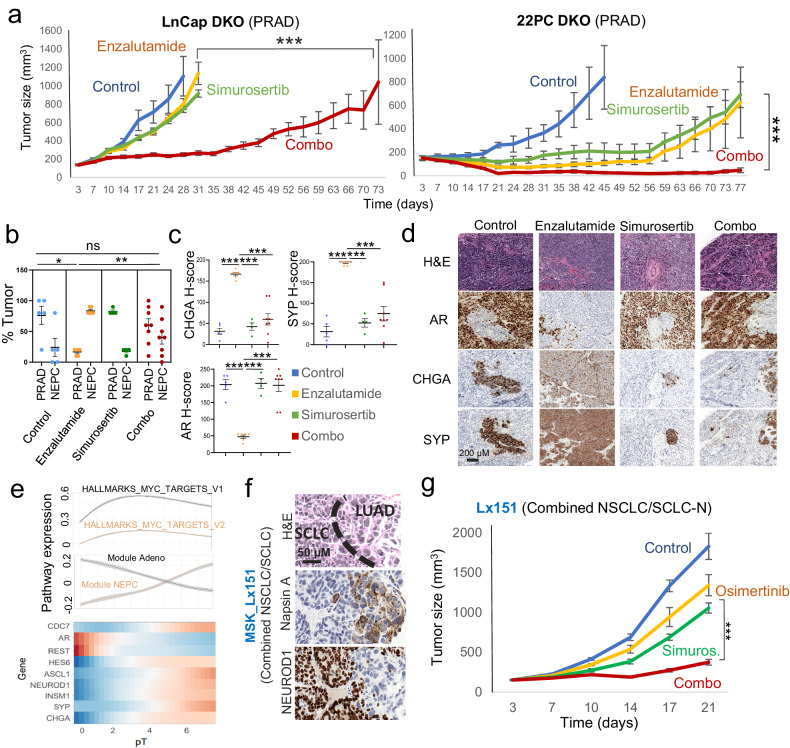
CDC7 inhibition suppresses NE transformation. **a** In vivo treatment of cell line xenografts for TP53/RB1-inactivated (DKO) LnCap/AR and 22PC cells with vehicle (*N* = 8 for LnCap/AR and *N* = 4 for 22PC) enzalutamide (*N* = 10, *N* = 6), simurosertib (*N* = 9, *N* = 4) or their combination (*N* = 10, *N* = 7). **b** Barplot showing the average ± standard deviation of the intratumoral percentages of adenocarcinoma and NE histology in tumors collected at endpoint for each treatment arm, including vehicle (*N* = 5), enzalutamide (*N* = 7), simurosertib (*N* = 6) and their combination (*N* = 4). Barplot showing the average ± standard deviation of H-score quantification (**c**) and representative images (**d**) for immunohistochemical assessment of the expression of androgen receptor (AR), synaptophysin (SYP) and chromogranin A (CHGA) staining in LnCap/AR tumors collected at endpoint for each experimental arm. **e** Trajectory analyses on single-cell transcriptomic data for the control and enzalutamide-treated DKO LnCap/AR tumors, illustrating changes in expression of gene of interest and in enrichment for gene signatures of interest. The lower panel consists of a heatmap of gene trends of select genes of relevance in NE transformation ordered by the putative transition from adenocarcinoma to NEPC in control and enzalutamide-treated cells from the in vivo treatment experiment in Fig. 3a. The top panel shows a spline fit of the average *Z*-score for GSEA pathways of interest. **f** H&E and IHC staining for markers of interest for the *EGFR*-mutant combined NSCLC/SCLC PDX tumor MSK_Lx151. **g** In vivo treatment of the MSK_Lx151 PDX with vehicle (*N* = 5), Osimertinib (*N* = 6), simurosertib (*N* = 5) or their combination (*N* = 7). For **a**–**c** and **j**, *p*-values were calculated using the Student’s *t* test (unpaired, heterogeneous variances, two-tailed). *p*-value legend: *<0.05, **<0.01, ***<0.001, ****<0.0001, ns not significant

To evaluate this hypothesis, we leveraged previously described prostate models of targeted therapy-induced adenocarcinoma-to-NE transformation.^3,18^ Double knock out (DKO) of *TP53* and *RB1* in the PRAD cell lines LnCap/AR and 22PC facilitates acquired resistance to AR-targeted therapy such as enzalutamide in vivo, together with a loss of epithelial features and increased NE marker expression.^3,18^ We treated xenografts of these models in immunocompromised mice with enzalutamide, simurosertib or both agents (Fig. 3a and Supplementary Fig. 4a). Consistent with our prior work,^3,18^ the DKO LnCap/AR xenografts showed resistance to enzalutamide (Treatment/Control (T/C) value of 72% at control arm experimental endpoint) and the DKO 22PC xenografts developed resistance to the drug after an initial sensitive period (Fig. 3a). Sensitivity to simurosertib monotherapy was similar to that observed for enzalutamide, with intrinsic resistance in the DKO LnCap/AR model (T/C values of 67% at control arm endpoint) and acquired resistance in the DKO 22 PC model (Fig. 3a). However, the combination treatment showed markedly prolonged efficacy in both models, with T/C values of 19 and 3% at control arm endpoint for DKO LnCap/AR and 22PC respectively, and a substantial delay in tumor relapse compared to either monotherapy (31 days versus 73 days for enzalutamide- and combo-treated tumors, respectively, for DKO LnCap/AR; and no relapse observed during the duration of the experiment for DKO 22PC; Fig. 3a). Histological assessment of the tumors collected at endpoint for each of the treatment arms could only be performed for the DKO LnCap/AR model, as no remaining tumor tissue could be found in the necropsy for the combo-treated DKO 22PC mice. Even in the untreated group, the DKO LnCap/AR tumors exhibited a mixed histological phenotype including PRAD and NEPC areas in each of the tumors (Fig. 3b). As expected,^3,18^ quantification of the PRAD and NEPC components showed a significant enrichment in NEPC component in the enzalutamide-treated tumors relative to untreated controls (average of 83% vs 24%, *p* = 0.015). This histologic shift was inhibited in the combination-treated group (NEPC in enzalutamide alone vs. the combination, 83% vs 40%, *p* = 0.005), with no significant differences in histologic representation in the combo versus control groups (*p* = 0.40, Fig. 3b). Consistently, immunohistochemical quantification of the NE markers chromogranin A and synaptophysin revealed an increased NE phenotype in enzalutamide-treated tumors, again reverted by the combination treatment (Fig. 3c, d). And conversely, AR protein expression was downregulated by enzalutamide treatment alone, but maintained in the tumors on the combination arm (Fig. 3c, d). Mice treated with the combination of simurosertib and enzalutamide had no overt signs of toxicity, including no significant weight loss compared to control mice, or mice treated with either single agent (Supplementary Fig. s4b).

We performed single-cell transcriptomic profiling on control and enzalutamide-treated tumors from the DKO LnCap/AR experiment (Fig. 3a–d) at endpoint. To study how NE transformation occurs in this model with higher granularity, we performed trajectory analyses modeling NE transdifferentiation (Fig. 3e). Further supporting the biological fidelity of these models, our analyses revealed positive and negative enrichment of previously described^12^ NEPC and adenocarcinoma gene modules, respectively, in cells along the trajectory (Fig. 3e). These occurred in parallel to other NE-transformation canonical features,^1^ such as downregulation of AR, inactivation of Notch signaling (illustrated by downregulation of *REST* and upregulation of *HES6*), upregulation of NE markers such as *NEUROD1, INSM1, SYP* and others (Fig. 3e), and consistent with our previous observations (Fig. 1c–f), CDC7 upregulation.

We also analyzed effects on a T-SCLC PDX derived from a patient with combined *EGFR*-mutant NSCLC/SCLC.^18^ This model, harboring a X187_splice TP53 mutation and RB1 deep deletion, retains both NSCLC ( ~ 5%) and SCLC (~95%) components, potentially mimicking an intermediate state of transformation (Fig. 3f).^18^ Treatment of this PDX model with osimertinib yielded limited efficacy, with a T/C value of 73% at control arm experimental endpoint; simurosertib monotherapy showed increased efficacy (T/C of 39%), and the combination outperformed any other treatment condition under assay, with a T/C value of 20% (Fig. 3g). Analysis of the LUAD marker TTF-1, NE markers synaptophysin and chromogranin A and morphology in the tumors collected at endpoint for each treatment arm did not show relevant differences (Supplementary Fig. s4c, d), suggesting that simurosertib might not be able to revert NE transformation after it has already occurred. No evident combinatorial toxicity with osimertinib and simurosertib vs. osimertinib alone were observed, as per mouse body weight assessment (Supplementary Fig. s4b).

These results suggest that CDC7 inhibition interferes with the acquisition of a NE phenotype in models of NE transformation. In combination with targeted therapy, simurosertib may be able to prevent NE transformation, although not revert it, and can substantially extend response in patients with tumors at high risk of NE transformation.

### CDC7 inhibition induces MYC degradation and suppresses NE transformation

**Fig. 4:**
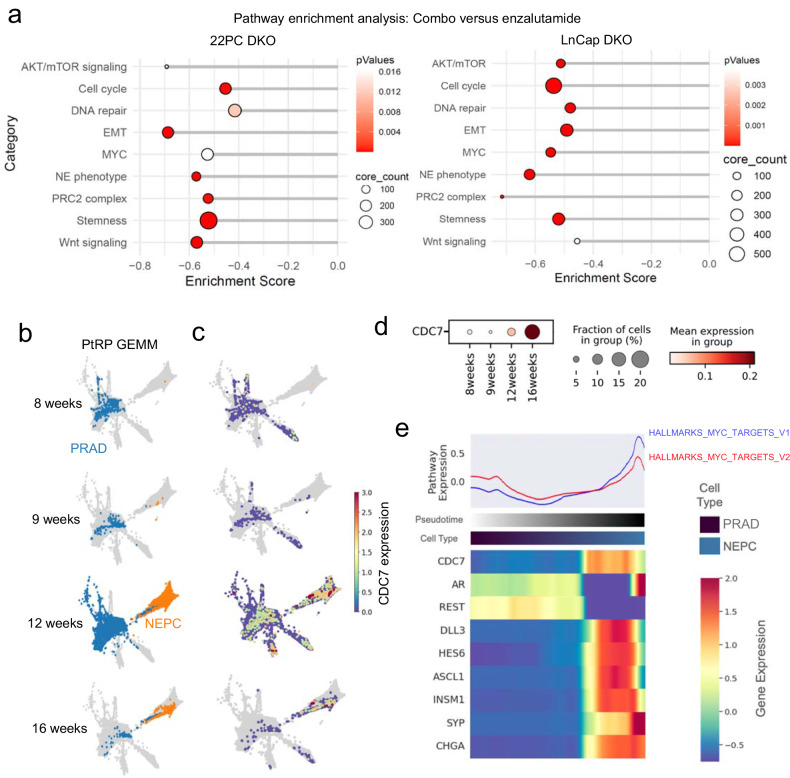
In combination with targeted therapy, CDC7 inhibition induces downregulation of genes involved in pathways associated to NE transformation. **a** Dotplots showing results for the pathway enrichment analysis on DEGs from combo- versus enzalutamide-treated tumor conditions in the transcriptomic data from TP53/RB1-inactivated 22PC and LnCap/AR xenografts treated in vivo and collected at an intermediate timepoint (day 17). Categorized pathways of interest, previously implicated in NE transformation,^1,2^ are shown. **b** Force-directed layouts (FDLs) for single-cell transcriptomic data in tumors collected during a time course experiment from a GEMM prostate NE transformation model (PtRP) described in ref. ^12^ showing increased NE transformation phenotype over time. FDLs are separated by time point, and cells undergoing NE transformation are labeled according to their trancriptomic profile resembling PRAD or NEPC. **c** FDLs separated by time point, with cells for each time point colored by CDC7 expression levels. **d** Dotplot showing the magnitude of CDC7 expression and percentage of CDC7-expressing cells at the different time points of the time course GEMM experiment. **e** Heatmap of gene trends of select genes of relevance in NE transformation ordered by the putative transition from adenocarcinoma to NEPC, with gene labels colored in the same way the aforementioned groups (scale gene trends of imputed expression, −0.5 to 2.0). The top panel shows a spline fit of the average *Z*-score for GSEA pathways of interest

To explore mechanisms by which CDC7 inhibition might interfere with NE transformation in tumors under selective pressure of targeted therapy, we performed bulk transcriptomic analyses on tumors collected at an intermediate time point (day 17) from our in vivo experiments with DKO LnCap/AR and 22PC (Fig. 3a). Differential gene expression (DGE) between the combo- versus enzalutamide-treated tumors for both models, and subsequent pathway enrichment analyses, showed downregulation of pathways previously implicated in NE transformation (Fig. 4a).^1,2,10,25^ These included regulatory pathways of cell cycle and DNA damage repair, stemness, epithelial-to-mesenchymal transition (EMT), the PRC2 epigenetic remodeling complex, as well as the AKT/mTOR, Wnt, and MYC pathways (Fig. 4a).

MYC in particular is a key stemness-associated transcription factor^26^ previously implicated in histological transformation,^1,27^ known to be a driver of SCLC tumors^28,29^ and also associated with resistance to targeted therapies.^30^ Based on its role as promoter of stem-like features, we hypothesized that it might function as an early actor in NE transformation, facilitating deprogramming and reprogramming of adenocarcinoma into a NE state. We performed single-cell transcriptomic trajectory analyses on a time course of tumor collections from a recently published^12^ genetically engineered mouse model (GEMM) of prostate cancer that recapitulates the adenocarcinoma-to-NEPC transition after prostate-specific deletion of the tumor suppressor genes *PTEN, RB1*, and *TP53* using CRE recombinase, Pten^−/−^Rb^−/−^Trp53^−/−^ (PtRP) (Fig. 4b–e), and leveraged our trajectory analyses for the DKO LnCap/AR model (Fig. 3e). In both models, we observed upregulation of multiple components of MYC gene signatures preceding the downregulation of *AR* and markers of Notch signaling (*REST*), together with the upregulation of Notch suppressors *DLL3* and *HES6*, and of NE markers including *INSM1, SYP* and *CHGA*, as well as *ASCL1 (*for the both models*)* and *NEUROD1 (*for the DKO LnCap/AR model*)* (Figs. 3e and 4e). Altogether, these results suggested an early role for MYC in NE transformation and prompted further investigation of whether suppression of MYC activity by CDC7 inhibition mechanistically contributed to abrogation of NE transformation.

**Fig. 5:**
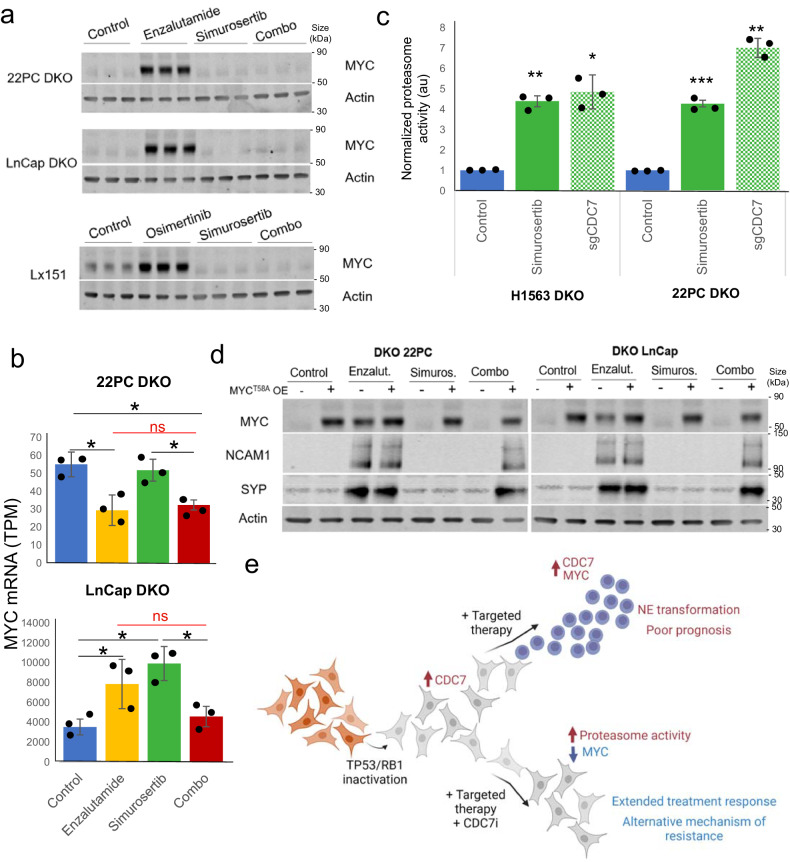
CDC7 inhibition-induced MYC degradation prevents NE transformation. **a** Western blots of tumors collected from our in vivo experiments in NE transformation models of prostate and lung (Fig. 3a, g) at an intermediate time point (day 17), assessing the expression of the transcription factor MYC. **a** Barplots showing CDC7 mRNA expression determined by RNAseq in transcripts per million (TPM) units in the tumors collected from our in vivo experiments in NE transformation models of prostate (Fig. 3a). **c** Barplots showing proteasome 20 s activity in TP53/RB1-inactivated LUAD (H1563) and prostate (22PC) cell lines with pharmacological (simurosertib) or genetic (CRISPR-Cas9 KO) CDC7 inhibition. Proteasome activity is shown as normalized by the control condition. **d** Western blots for isogenic cell lines of TP53/RB1-inactivated 22PC and LnCap/AR prostate models of NE transformation, with or without MYC^T58A^ exogenous overexpression. Cells were treated with enzalutamide, simurosertib or their combination in vitro for 7 days, and cells were collected for protein extraction, to assess NE marker (NCAM1, SYP) and MYC expression. **e** Schematic model of CDC7 inhibition in NE transformation. Briefly, TP53/RB1 inactivation leads to increased expression of and dependency on CDC7 in lung and prostate adenocarcinomas. Targeted therapy can promote NE transformation, involving MYC induction in the process, and leading to poor prognosis. However, CDC7 inhibition in combination with targeted therapy activates proteosomal degradation of MYC. Without the action of MYC, NE transformation and adaptation to therapy is constrained, leading to extended response to treatment. Tumors may eventually acquire resistance to therapy via alternative mechanisms of resistance. Created with BioRender.com. For **b** and **c**, *p*-values were calculated using the Student’s *t* test (unpaired, heterogeneous variances, two-tailed). *p*-value legend: *<0.05, **<0.01, ***<0.001, ns not significant

Characterization of MYC protein expression by western blot in tumors from the previously described in vivo treatment experiments with the DKO LnCap/AR and 22PC prostate models of transformation (Fig. 3a), as well as with the MSK_Lx151 lung combined NSCLC/SCLC model (Fig. 3f, g), revealed strong upregulation of MYC expression by targeted therapy in all 3 models, which was suppressed in the combo-treated tumors (Fig. 5a). Assessment of MYC mRNA levels did not track with protein levels in these contexts (Fig. 5b), suggesting that combo treatment-induced downregulation of MYC was not transcriptionally regulated. It is known that MYC function is tightly controlled by regulation of protein stability via modulation of proteasomal degradation.^31^ Evaluation of proteasome 20 s activity in *TP53/RB1*-inactivated H1563 and 22PC indicated significant induction of proteasomal activity by simurosertib or CDC7 CRISPR KO (Fig. 5c), consistent with MYC suppression by simurosertib being driven by MYC proteasomal degradation. To assess whether CDC7 inhibition-induced MYC degradation could be preventing NE transformation, we performed rescue experiments in the two prostate transformation models (DKO LnCap/AR and 22PC) by ectopically inducing expression of MYC^T58A^, a proteasomal degradation-resistant MYC isoform (Fig. 5d). In both models, untreated control cells showed limited expression of the NE markers NCAM1 and SYP, which remained unchanged upon MYC^T58A^ overexpression. Consistent with in vivo observations (Fig. 3b–d), enzalutamide treatment triggered the expression of NE markers in these cells, again irrespective of MYC^T58A^ overexpression. In combo-treated cells, simurosertib suppressed expression of NE markers by enzalutamide in the absence of MYC^T58A^, while MYC^T58A^ overexpression was able to fully restore NE marker induction (Fig. 5d). As an alternative approach to induce MYC downregulation, we treated these models as well with the combination of enzalutamide and MRT-2359, a GSPT1-directed molecular glue degrader with the ability to downregulate MYC expression^32^ (Supplementary Fig. s4e). Similarly to simurosertib, MRT-2359 treatment-induced MYC downregulation and loss of enzalutamide-induced NE markers.

Taken together, these results support a model in which inactivation of TP53 and RB1 lead to a state primed for histological transformation, characterized by increased expression of CDC7. Treatment with targeted therapy further increases expression of and dependency on CDC7; CDC7 in turn inhibits proteasomal activity, stabilizing MYC and facilitating NE transformation. CDC7 inhibition by simurosertib restores proteasome activation and MYC degradation; in a low-MYC state, tumor cells are unable to progress through NE transformation, leading to extended treatment response (Fig. 5e).

### CDC7 inhibition sensitizes NE tumors to chemotherapy

**Fig. 6:**
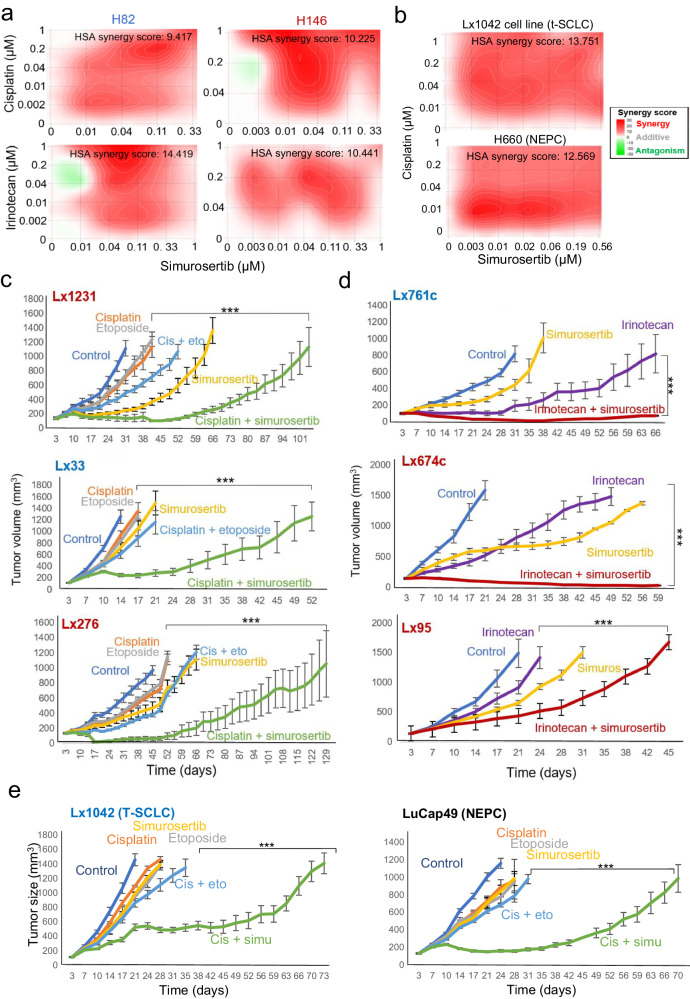
CDC7 inhibition dramatically sensitizes de novo SCLC PDX to first and second line chemotherapy. In vitro synergy assays in H82 (SCLC-N, left) and H146 (SCLC-A, right) cell lines of the combination of simurosertib and cisplatin or irinotecan (**a**), or in Lx1042 (T-SCLC) and H660 (NEPC) with the combination of simurosertib and cisplatin (**b**), with average synergy score displayed, as assessed by Highest Single Agent (HSA) method, and calculated using SynergyFinder. Representative plots are shown. In vivo treatments of high (Lx1231, SCLC-A), intermediate (Lx33, SCLC-N) and low (Lx276, SCLC-A) CDC7-expressing PDX derived from treatment-naïve tumors (**c**), of high (Lx761c, SCLC-N), intermediate (Lx674c, SCLC-A) and low (Lx95, SCLC-A) CDC7-expressing PDX derived from tumors progressed on chemotherapy (**d**), and of NE-transformed PDX (Lx1042 and H660)(**e**), to compare the efficacy of the combination of simurosertib with cisplatin versus that of cisplatin and etoposide (chemotherapy-naïve PDX, including NE-transformed PDX) or of simurosertib with irinotecan versus irinotecan alone (progressed on treatment PDX). *p*-values for (**a**), (**d**), and (**e**) were calculated using the Student’s t-test (unpaired, heterogeneous variances, two-tailed). *p*-value legend: *<0.05, **<0.01, ***<0.001

Previous reports have suggested a role for CDC7 in chemoresistance in a variety of settings, including breast, ovarian, pancreatic and non-SCLC tumors.^16^ A recent report showed that in vitro-induced chemoresistance can be reverted by the CDC7 inhibitor XL413 in SCLC cell lines.^33^ We sought to further assess whether cytotoxic therapies for SCLC might be augmented by CDC7 inhibition. We observed that CDC7 knockout induced sensitivity to cisplatin in two cell lines representing distinct SCLC subtypes (H82, SCLC-N and H146, SCLC-A, Supplementary Fig. s5a) and that the combination of simurosertib with either cisplatin or irinotecan, used in the first and second line treatment of SCLC patients, respectively, exerted synergistic effects in vitro in both models (Fig. 6a), associated with induction of apoptosis (Supplementary Fig. s5b). Similarly, the combination of cisplatin and simurosertib showed synergistic effects in a cell line derived from a T-SCLC PDX, Lx1042, and in H660, a NEPC cell line (Fig. 6b). After characterizing the CDC7 protein expression levels in our SCLC PDX platform including PDX derived from treatment-naïve and treated patients (Supplementary Fig. s5c), we selected 3 treatment-naïve PDX models with high (H-score>150), intermediate (150 > H-score>50) and low (H-score<50): Lx1231 (SCLC-A), Lx33 (SCLC-N), and Lx276 (SCLC-A), respectively. In all 3 models, the combination of simurosertib and cisplatin outperformed cisplatin and etoposide (Fig. 6c), with T/C values at control experimental endpoint of 13% vs 44% for Lx1231, 20% vs 49% for Lx33, and 7% vs 39% for Lx276, respectively (Fig. 6c). Similarly, we selected a high, intermediate and low CDC7-expressing PDX derived from chemotherapy-progressed tumors: Lx761c (SCLC-N), Lx674c (SCLC-A) and Lx95 (SCLC-A), respectively (Fig. 6d). Again, for all 3 models, the combination of irinotecan and simurosertib showed superior efficacy to that of irinotecan alone, with T/C values of 2% vs 24% for Lx761c, 6% vs 33% for Lx674c, and 29% vs 61% for Lx95, respectively (Fig. 6d). In all cases, no evident additional toxicity was attributable to combination treatments over that of chemotherapy alone (cisplatin or irinotecan) as assessed by serial body weight measurements (Supplementary Fig. s5d). In vivo treatments of the T-SCLC PDX Lx1042 (CDC7 H-score: 250) and NEPC PDX LuCap49 (CDC7 H-score: 210) likewise demonstrated sensitivity to the cisplatin and simurosertib combination, outperforming response to any single agent, and to the clinically relevant combination of cisplatin and etoposide, with T/C values at control experimental endpoint of 36% vs 59% for Lx1042, and of 14% vs 59% for LuCap49, respectively (Fig. 6e).

In order to test whether our results may be extendable to other NE tumor types, we leveraged again the CCLE transcriptomic dataset to assess the correlation between CDC7 mRNA expression and NE phenotype. We observed significant positive correlations of CDC7 expression with an extensively used NE gene signature, as well as with key NE markers (Supplementary Fig. s5e), where cell lines derived from tumors with NE features showed high CDC7 expression, suggestive of an association of high CDC7 expression and NE tumor phenotype beyond SCLC and NEPC. Indeed, when we performed in vitro synergy assays in cell lines from another NE subtype of lung cancer (Supplementary Fig. s5f), large cell neuroendocrine carcinoma (LCNEC), we observed strong synergy between cisplatin and simurosertib in this additional NE tumor type.

Together, these results nominate simurosertib as a potent chemosensitizing agent for de novo and transformed NE lung and prostate tumors, and suggest these results might be extendable to additional NE tumor types.

Bulk transcriptomic analysis on the H146 (SCLC-A) cell line in vitro and Lx33 (SCLC-N) in vivo treated with cisplatin and simurosertib, as well as on the Lx761c (SCLC-N) in vivo treated with irinotecan and simurosertib (Supplementary Fig. s6a) consistently revealed global downregulation of genes involved in MYC signaling in the combo-treated tumors versus the chemotherapy monotherapy-treated tumors, similar to what was observed in the NE transformation models treated with simurosertib (Fig. 4a). Prior reports have suggested a potential role for MYC in chemotherapy resistance,^34^ describing MYC upregulation in SCLC cell lines and PDX upon acquisition of chemotherapy resistance, reporting a MYC transcriptional signature enriched in tumor biopsy and circulating tumor cell (CTC)-derived SCLC PDX from patients with chemoresistant disease^35^ and characterizing a SCLC GEMM with MYC^T58A^ overexpression able to acquire resistance to chemotherapy dramatically faster than other SCLC GEMM with no or low MYC expression.^28,29^ Analysis of MYC protein expression in our in vivo PDX treatments with simurosertib and chemotherapy indicated that MYC protein levels are upregulated in tumors treated with cisplatin or irinotecan, but suppressed in tumors treated with simurosertib, alone or in combination with either chemotherapeutic agent (Supplementary Fig. s6b–d). Assessment of MYC mRNA expression in models receiving these treatments in vitro or in vivo did not reveal consistent downregulation of MYC mRNA by simurosertib (Supplementary Fig. s6e, f). Consistent with what we observed in adenocarcinoma cell lines (Fig. 5c), genetic (KO) or pharmacological inhibition of CDC7 again induced the activation of the proteasome (Supplementary Fig. s6g). These results suggested that CDC7 inhibition may induce MYC degradation, thus hindering the development of chemoresistance.

Next, we aimed to study whether MYC overexpression induces chemoresistance. MYC^T58A^ overexpression did not lead to increased resistance to chemotherapeutic agents in isogenic SCLC cell lines (Supplementary Fig S6h, i), suggesting that MYC may not induce immediate chemoresistance. To study whether MYC overexpression may promote the acquisition of chemotherapy resistance at a longer term, we overexpressed wild type MYC and MYC^T58A^ in two SCLC cell lines with limited or no endogenous MYC expression (H146 and H69, Supplementary Fig s6h) that were treated with cisplatin alone or in combination with simurosertib (Supplementary Fig s6j). In the absence of treatment, neither MYC isoform caused differences in proliferation. The control cell lines treated with cisplatin would eventually adapt to the drug and start regrowing after a period of growth arrest (Time ~91 days for H146 and ~84 days for H69), but no regrowth was observed for the control cell lines treated with cisplatin and simurosertib during the duration of the experiment. The MYC^T58A^-overexpressing cell lines initiated regrowth significantly earlier than control cell lines(Time ~63 days for H146 and ~56 days for H69). When these were treated with the combination of cisplatin and simurosertib, cells suffered a slight delay until regrowth (Time ~70 days for H146 and ~63 for H69) but still started regrowth significantly earlier than their control counterparts. Lastly, no regrowth was observed for the MYC KO cell lines during the duration of the experiment, for either cisplatin or combination treatment conditions (Supplementary Fig s6j). These results suggest that, even if MYC does not directly drive resistance to chemotherapy, its expression may promote the eventual development of drug resistance. Treatment with the CDC7 inhibitor simurosertib in combination with chemotherapy may lead to proteasome overactivation and subsequent MYC proteasomal degradation, thus hindering the appearance of drug resistance, that may eventually develop through MYC-independent mechanisms (Supplementary Fig s6j).

## Discussion

Histological transformation represents a complex mechanism of resistance to targeted therapy, involving deep epigenetic reprogramming and associated with altered activity of multiple oncogenic signaling pathways.^1,2^ Recent molecular and functional characterizations in GEMM and clinical specimens have refined the list of key molecular contributors to lineage plasticity driving NE transformation, including inactivation of *TP53* and *RB1*, activation of the AKT/mTOR pathway and induction of MYC.^1,2,6,10,11^ The difficulty of pharmacologically targeting these oncogenic drivers in a potent and durable manner has precluded development of clinical strategies to prevent histological conversion, even while identifying individuals at high risk of transformation.^6^ The development of therapeutic approaches to prevent NE transformation is thus an unmet clinical need, of particular relevance due to the association of this mechanism of resistance with strikingly poor prognoses.^7^

In the present work, we identify CDC7 inhibition as a potential therapeutic approach in two different settings: inhibition of NE transformation from adenocarcinomas on targeted therapy, and treatment of NE carcinomas, either de novo or following histological transformation. This cell cycle kinase involved in DNA replication and DNA damage response is upregulated during transformation and highly expressed in NE carcinomas of the lung and prostate, consistent with the high proliferative indices and genomic instability of these tumors.^1,36–38^ Our data suggest that the interplay between TP53, RB1, and CDC7 may be mediated by *CDC7* gene regulation by TP53 and the RB1 downstream effector E2F1. These results are in resonance with previous literature describing gain-of-function mutations in TP53 that lead to enhanced CDC7-dependent replication initiation in LUAD,^15^ enrichment for RB1 and TP53 mutations in CDC7-high LUAD,^23^ and increased sensitivity to CDC7 inhibition in TP53-mutant breast carcinomas.^39^ We extend these observations, demonstrating that *TP53-* and *RB1*-deficient adenocarcinomas of the lung and prostate have increased sensitivity to CDC7 inhibition, providing a rationale to target CDC7 as a means to constrain histological transformation. However, the concrete molecular mechanism by which CDC7 expression is regulated by TP53 and RB1, and why their inactivation leads to increased dependency on this kinase remain to be elucidated.

CDC7 inhibition markedly extended response to targeted therapy in two prostate in vivo models of adenocarcinoma-to-NE transformation and a combined NSCLC/SCLC PDX derived from a T-SCLC tumor. The combination treatment was able to induce full regressions in one of the prostate models, and to prevent progression of NE transformation in the second, in sharp contrast to the effects of enzalutamide as monotherapy. Importantly, CDC7 inhibition was unable to revert NE transformation in the minor NE component present at baseline in this prostate model, or in the combined histology SCLC PDX showing a major NE component at baseline. These results suggest that CDC7 inhibition might be able to prevent, but not reverse, NE transformation.

Our data point to MYC stabilization by proteosomal inhibition as a potential mechanism by which CDC7 activity could promote lineage plasticity. MYC, a transcription factor related to stemness^26^ previously implicated in histological transformation^1,27^ and as a driver of the NE phenotype,^11,25^ was induced at the protein level during NE transformation and suppressed by CDC7 inhibition, concurrent with suppression of NE transformation. CDC7 modulation had no consistent effect on MYC gene transcription, but rather appeared to affect MYC protein stability via modulating proteasomal degradation. Exogenous expression of the degradation-resistant MYC^T58A^ isoform was able to rescue the NE transformation phenotype, even in the presence of CDC7 inhibition. This observation may be useful to identify patients at high risk of transformation that may not respond to CDC7 inhibition if their tumor harbors MYC genomic alterations that render it resistant to proteasomal degradation. Altogether, these results provide insight into CDC7 mechanisms of action and further highlight the essentiality of MYC in NE transformation.

The proteasome is involved in DNA repair processes during the cell cycle,^40^ with its inhibition sensitizing to different chemotherapeutic DNA damaging agents in different tumor settings.^41^ Analogously, previous results confirmed by our own suggest that CDC7 is also involved in DNA damage repair, as its inhibition suppresses homologous recombination repair and sensitizes to chemotherapy.^16^ We hypothesize that the mutational stress triggered by the inhibition of CDC7 may lead to the activation of alternative DNA damage repair pathways involving the proteasome, inducing overactivation of the proteasome as a compensation mechanism.

Beyond its role in NE transformation, recent results from our group nominate MYC as a driver of adenocarcinoma-to-squamous transformation,^27^ suggesting that MYC might have a generalized role in promoting plasticity, inducing developmental reversion to a stem-like state permissive for histological transdifferentiation across multiple lineages. This would suggest that the contribution of MYC to NE transformation may occur in early stages of the process, which would be consistent with our observation that CDC7 inhibition is able to prevent, but not revert, NE transformation.

While not previously identified as a factor in lineage plasticity, CDC7 has been reported as a contributor to chemoresistance across tumor types including breast, ovarian, pancreatic and non-SCLC.^16^ A recent report showed that in vitro-induced chemoresistance can be reduced by the CDC7 inhibitor XL413 in SCLC cell lines.^33^ To more deeply profile the potential of CDC7 inhibition in high-grade NE cancers, we treated an array of PDX derived from de novo and transformed SCLC and NEPC with the combination of cisplatin and simurosertib (treatment-naïve PDX) or irinotecan and simurosertib (pre-treated PDX). Both combinations showed consistent efficacy, suppressing tumor growth and substantially extending response to chemotherapy, independent of basal CDC7 expression levels, and without evident additional toxicity. Complete tumor regressions were observed in 2 out of 8 PDX models tested. Additionally, analyses of publicly available datasets indicated that upregulation of CDC7 mediated by TP53/RB1 inactivation may occur beyond prostate and lung malignancies, and particularly in those tumors with a NE phenotype, suggesting that CDC7 inhibition may be an interesting therapeutic approach in combination with chemotherapeutic agents in many other tumor types, as we have demonstrated in another NE lung tumor type, LCNEC. These results support testing of CDC7 inhibitors such as simurosertib as chemosensitizing agents for the treatment of patients with high-grade NE carcinomas.

In sum, our data nominate CDC7 as a therapeutic target (1) to constrain plasticity and NE transformation as a mechanism of acquired resistance to targeted therapies in lung and prostate cancers; and (2) to induce sensitivity to cytotoxic therapy and extend response to treatment in lung and prostate NE tumors. The availability of clinically active CDC7 targeted inhibitors underscores the potential for rapid translation of these findings to patients at risk.

## Materials and methods

### Cell lines, plasmids, virus production, and transduction

Please refer to the Supplementary Materials and Methods section for information of cell lines, plasmids and transduction methods used in the present work.

### CRISPR-Cas9 screen

For a detailed description of our CRISPR-Cas9 screen, please refer to the Supplementary Materials and Methods section.

### Monotherapy cytotoxicity assay

Cytotoxic assays were performed as described.^42^ A total of 1,000 cells/well were seeded in 96-well plates and treated with the drugs/doses described for 96 hours. Viability was assessed with the CellTiter-Glo 2.0 Assay (Promega, G9242) as indicated by manufacturer.

### Synergy assays

Cells were seeded in 96-well plates (1000 cells/well) and treated with the interval of concentrations of cisplatin or simurosertib for 5 days. Then, cell viability was assessed with CellTiter-Glo 2.0 Assay (Promega, G9242) and normalized to the untreated wells. Synergy was calculated using the ZIP method using the SynergyFinder web application (2.0).^43^

### Proteasome activation assay

Proteasome activation in vitro was assessed with the Proteasome 20S Activity Assay Kit (#MAK172-1KT, Sigma), following the manufacturer’s instructions. The fluorometric proteasome 20 S assay kit is a homogeneous fluorescent assay using LLVY-R110 as a fluorogenic indicator for proteasome activities. Cleavage of LLVY-R110 by proteasome generates strongly green fluorescent R110. R110 signal was measured fluorimetrically at 525 nm with excitation at 490 nm. Untreated or simurosertib pre-treated 80,000 (H1563 and 22PC) or 300,000 (H82 and H146) cells were seeded in technical replicates (wells) from 96-well plate. Following addition of the 10x kit reagent, plates were incubated in a 5%CO_2_ incubator at 37 °C for 4 h in the dark. After that, 100 uL of Proteasome Assay Loading Solution was added per well, followed by 2 h of incubation and fluorescence measurement.

### Immunoblot

Protein extraction and western blot were performed as previously described^44^ from frozen cell pellets or flash-frozen tumor samples using RIPA lysis buffer with 1× HALT protease inhibitor cocktail (Thermo, # 78446). Cell pellets were resuspended in five volumes of cold lysis buffer and incubated on ice for 30 min. Lysates were clarified by centrifugation at 20,000×*g* for 10 min at 4 °C. For information on antibodies used, please refer to Supplementary Materials and Methods.

### Promoter reporter assays

A Promoter reporter clone for the human CDC7 gene (HPRM35138-LvPG04, Genecopoeia) was used in combination with a GAPDH positive control clone (GAPDH-LvPG04, Genecopoeia) and a negative control clone (NEG-LvPG04, Genecopoeia). Such clones were purchased in a lentiviral vector (LvPG04). Promoter reporter assays were performed as specified by manufacturer using the Secrete-Pair Gaussia Luciferase Dual and Single Luminescence Assay Kits (LF032, Genecopeia), where signal from constitutively secreted alkaline phosphatase activity was used to normalized *CDC7* promoter-dependent Gaussia Luciferase activity.

### Apoptosis assays

Cells were seeded in 6 well plates and treated with cisplatin (1uM), irinotecan (2uM), simurosertib (100 nM for H82, 350 nM for H146) or their respective combinations for 96 h. Next, cells were washed twice with PBS and stained with anti-Annexin V and propidium iodide for 30’ at room temperature. Cell death was determined by flow cytometry using a LSRFortessa™ Cell Analyzer.

### In vivo treatments

For detailed description of the in vivo experiments performed in this work, please refer to the Supplementary Materials and Methods section. All animal experiments were approved by the Memorial Sloan Kettering Cancer Center (MSKCC) Animal Care and Use Committee (#13-07-007).

### RNA extraction

Frozen tissues or cell pellets were weighed and homogenized in RLT and nucleic acids were extracted using the AllPrep DNA/RNA Mini Kit (QIAGEN, #80204) according to the manufacturer’s instructions. RNA was eluted in nuclease-free water.

### Computational analyses

For detailed computational methods for the analyses of our bulk RNAseq, ATACseq and single-cell RNAseq, please refer to the Supplementary Methods sections.

### Clinical samples

All study subjects had provided signed informed consent for biospecimen analyses under an Institutional Review Board-approved protocol. Metastatic prostate cancer samples were collected as part of the Prostate Cancer Donor Program at the University of Washington. Tissue microarrays sampling PRAD and NEPC]formalin fixed paraffin embedded tissues were used in this study.

### Statistical analyses

Comparisons between two groups were performed using paired or unpaired two-tailed Student’s *t* test, as indicated in Figure legends. A *p* value < 0.05 was considered statistically significant (*p ≤ 0.05, **p ≤ 0.01 and ***p ≤ 0.001). *N* indicates the number of biological replicates, all bars within the graphs represent mean values, and the error bars represent SEMs or standard deviation, as indicated in the Figure legend. All in vitro experiments were replicated a minimum of 3 times (biological replicates). All western blots have been replicated a minimum of two times (biological replicates). Please refer to previous sections for detailed statistical analyses of the bioinformatic data.

### Supplementary information

Supplementary Material

Data S1

Data S2

## Data Availability

All data, code, and materials used in the analysis is available upon reasonable request. Whole-tumor RNAseq datasets generated in the present manuscript are available at GSE252569. Single-cell RNAseq dataset generated in the present manuscript is available at GSE265887. Lung cancer patient-derived xenograft models will be provided upon MTA agreement.
