# Supplementary material for: CDC7 inhibition impairs neuroendocrine transformation in lung and prostate tumors through MYC degradation
Source: Signal Transduct Target Ther. 2024 Jul 26;9:189. doi: 10.1038/s41392-024-01908-y (PMC11272780; doi:10.1038/s41392-024-01908-y)
Supplement: Supplementary file 1 — Supplementary Material [file 41392_2024_1908_MOESM1_ESM.docx]

Supplementary Materials for

CDC7 inhibition impairs neuroendocrine transformation in lung and prostate tumors through MYC degradation

**Authors:** Alvaro Quintanal-Villalonga*, Kenta Kawasaki^±^, Esther Redin^±^, Fathema Uddin^±^, Swanand Rakhade^±^, Vidushi Durani, Amin Sabet, Moniquetta Shafer, Wouter R Karthaus, Samir Zaidi, Yingqian A. Zhan, Parvathy Manoj, Harsha Sridhar, Dennis Kinyua, Hong Zhong, Barbara P. Mello, Metamia Ciampricotti, Umesh K. Bhanot, Irina Linkov, Juan Qiu, Radhika A. Patel, Colm Morrissey, Sanjoy Mehta, Jesse Barne, Michael C. Haffner, Nicholas D. Socci, Richard P. Koche, Elisa de Stanchina, Sonia Molina-Pinelo, Sohrab Saleh, Helena A. Yu, Joseph M. Chan, Charles M. Rudin*.

Correspondence to: [quintaa1@mskcc.org,](mailto:quintaa1@mskcc.org,) rudinc@mskcc.org

**This PDF file includes:**

Figures. S1 to S6

Supplementary Materials and Methods

**Other Supplementary Materials for this manuscript include the following:**

Data S1 to S2:

- Data S1: Gene list with guide RNAs depleted from the CRISPR-Cas9 KO screen
- Data S2: TP53 and RB1 mutations in the publicly available clinical datasets where CDC7 expression was analyzed

**SUPPLEMENTARY FIGURES**

**
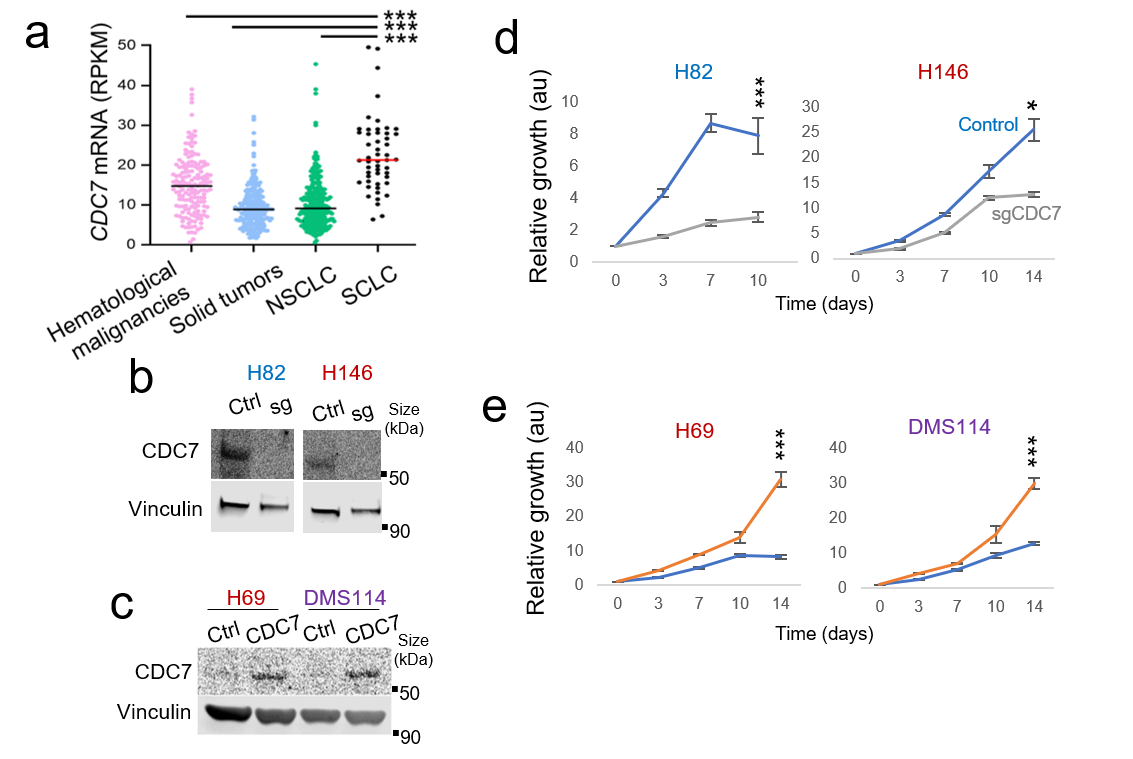
**

**
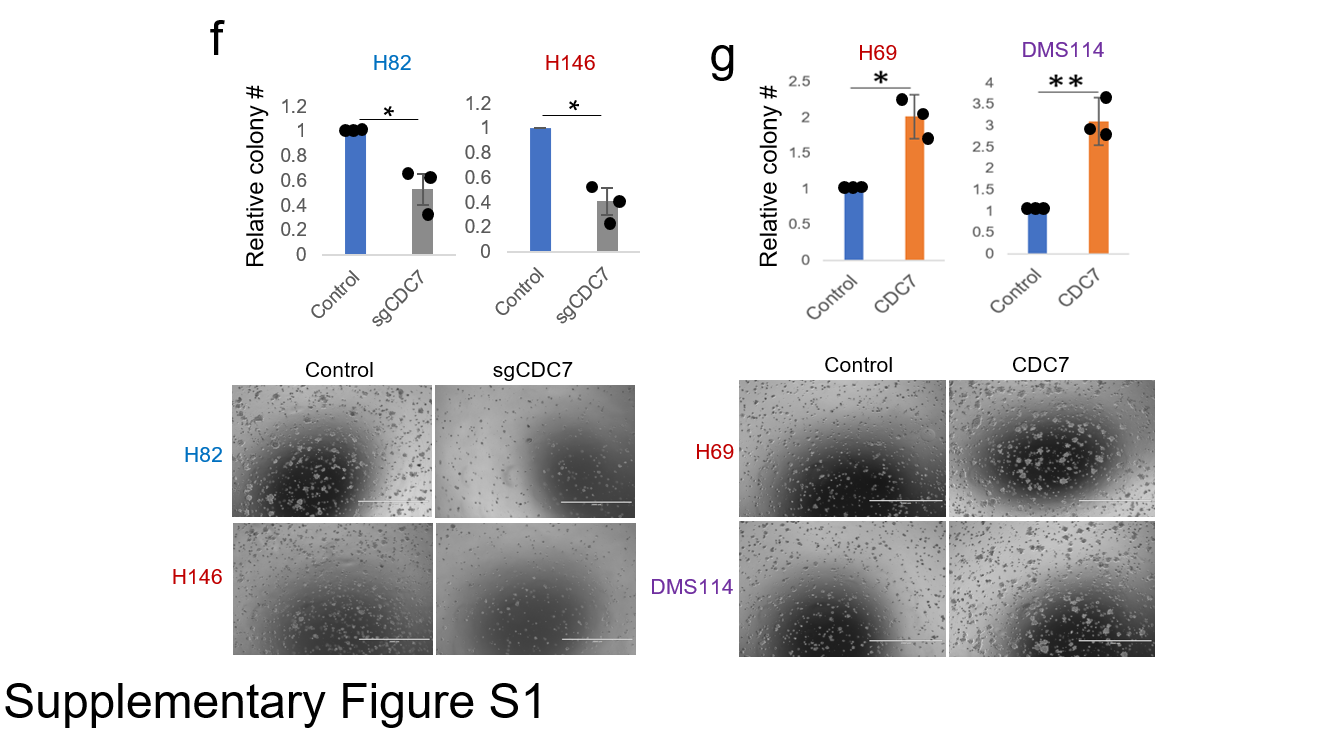
**

**Figure S1. Oncogenic role of CDC7 in SCLC.** (a) CDC7 mRNA expression in RPKM units in cancer cell lines from the Cancer Cell Line Encyclopedia (CCLE)^1^, categorized as cell lines derived from hematological malignancies, solid tumors, non-SCLC (NSCLC) and SCLC. Western blots showing CDC7 CRISPR-Cas9 KO in H82 (SCLC-N) and H146 (SCLC-A) cell lines (b) or CDC7 overexpression in H69 (SCLC-A) and DMS114 (SCLC-Y) (c). Growth curves for CDC7 KO (d) and CDC7 overexpressing (e) cell lines shown in (b) and (c), respectively. Proliferation is shown as relative growth normalized to day 0. Soft agar colony formation assay for CDC7 KO (f) and CDC7 overexpressing (g) cell lines shown in (b) and (c), respectively, and representative colony images. Colony number is shown as relative quantification, normalized to control condition. Experiments from D-G were reproduced a minimum of 3 times and a representative biological replicate is shown. P-values were calculated using the Student’s t-test (unpaired, heterogeneous variances, two-tailed). P-value legend: *<0.05, **<0.01, ***<0.001.


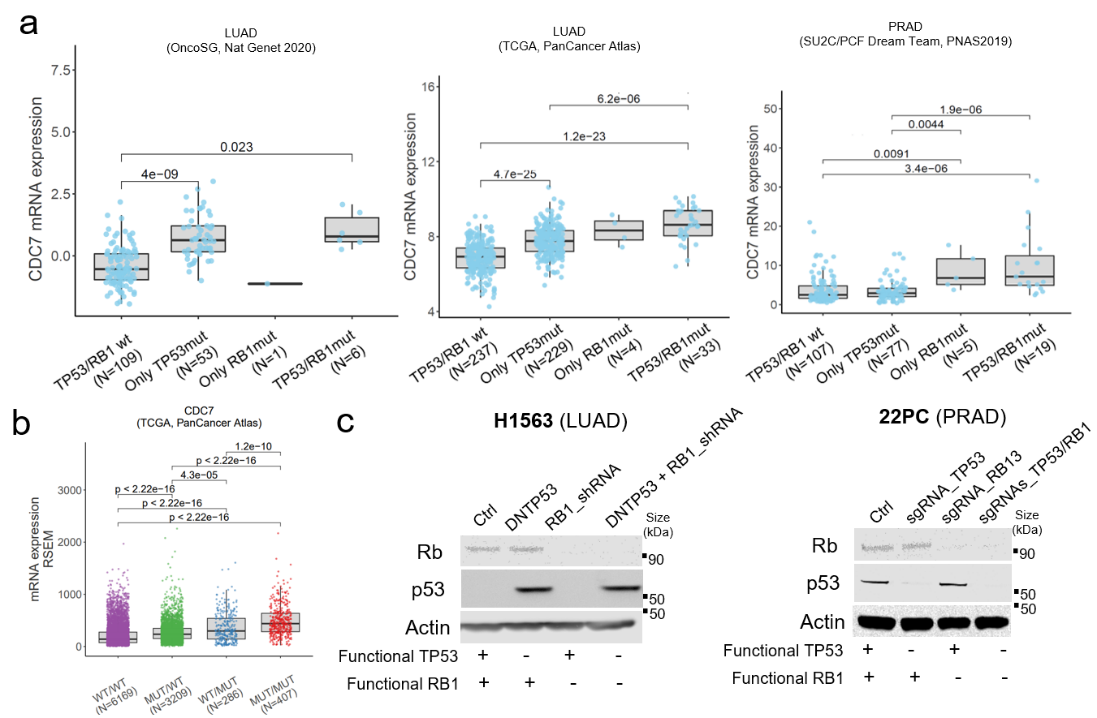

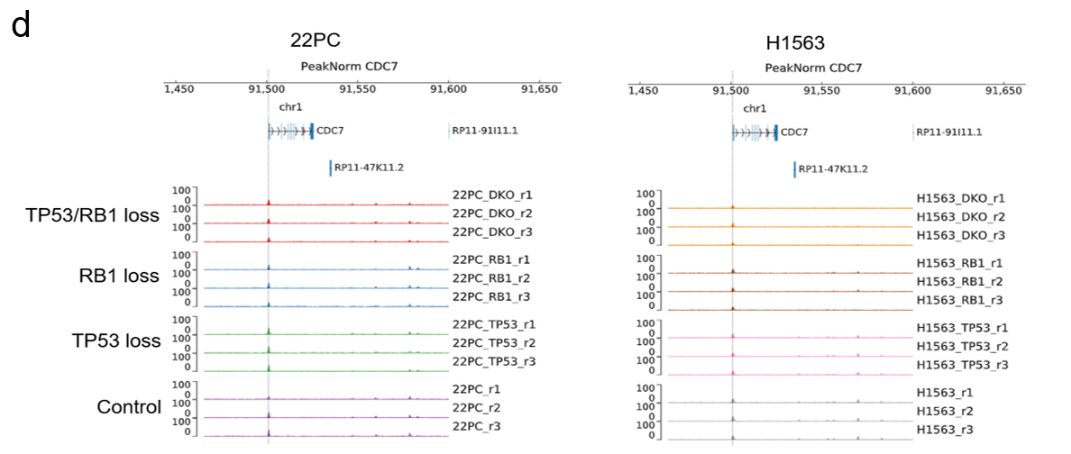


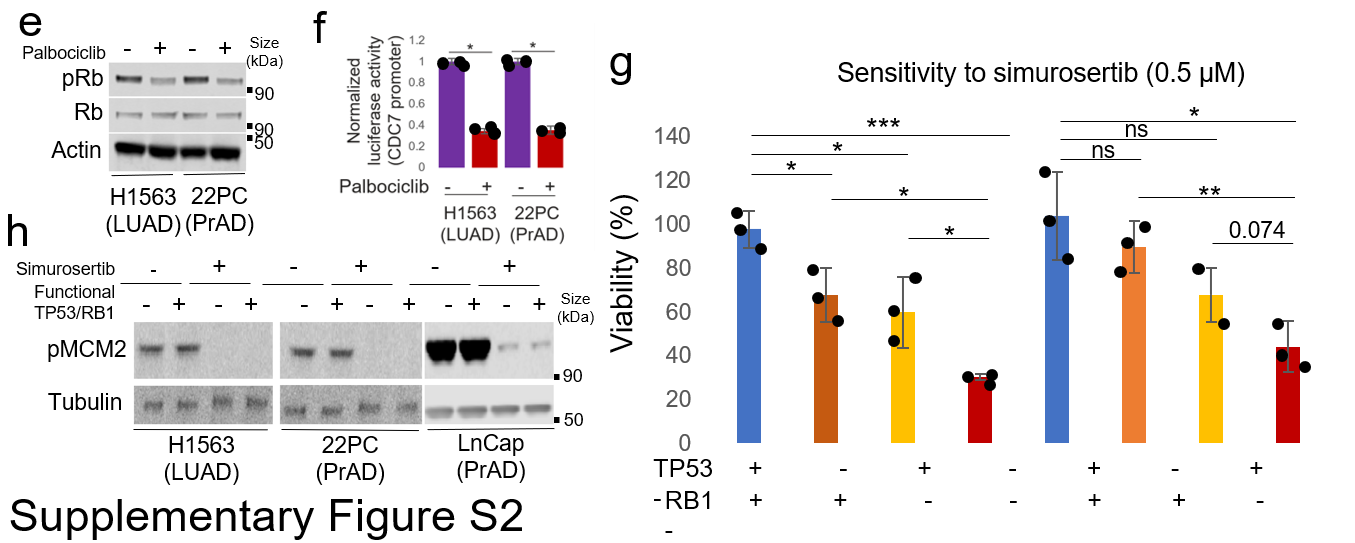


**Figure S2. Dysregulation of CDC7 and sensitivity to simurosertib.** (a) CDC7 mRNA expression in adenocarcinoma clinical specimens, categorized by *TP53/RB1* mutational status. Data obtained from LUAD TCGA (n=237 wild type (wt), 33 mutated), LUAD OncoSG (n=109 wt, 6 mutated)^2^ and PRAD SU2C/PCF Dream Team (n=107 wt, 19 mutated)^3^. (b) CDC7 mRNA expression in tumoral clinical specimens from the PanCancer TCGA integrative cohort, categorized by *TP53/RB1* mutational status. (c) Western blot showing P53 expression in our isogenic LUAD and PRAD cell lines with TP53 and/or RB1 inactivation. (d) DNA accessibility ATACseq data from isogenic control and TP53/RB1-inactivated H1563 and 22PC isogenic cell lines. The transcription start site for the CDC7 gene is indicated. (e) Western blot showing reduced levels of phospho-Rb (pRb) after treatment with pabociclib 1µM for 48 hours. (f) CDC7 promoter reporter assays showing downregulation of CDC7 promoter activity upon treatment with Palbociclib. (g) Plot showing a representative biological replicate of an experiment assessing viability of control and TP53- and/or RB1-inactivated H1563 and 22PC cells treated with 0.5 µM simurosertib. (h) Western blot showing pMCM2 expression in control and TP53- and/or RB1-inactivated H1563, 22PC and LnCap cells treated with 0.5 µM simurosertib. P-values for (e) and (h) were calculated using the Student’s t-test (unpaired, heterogeneous variances, two-tailed). P-value legend: *<0.05, **<0.01, ***<0.001.


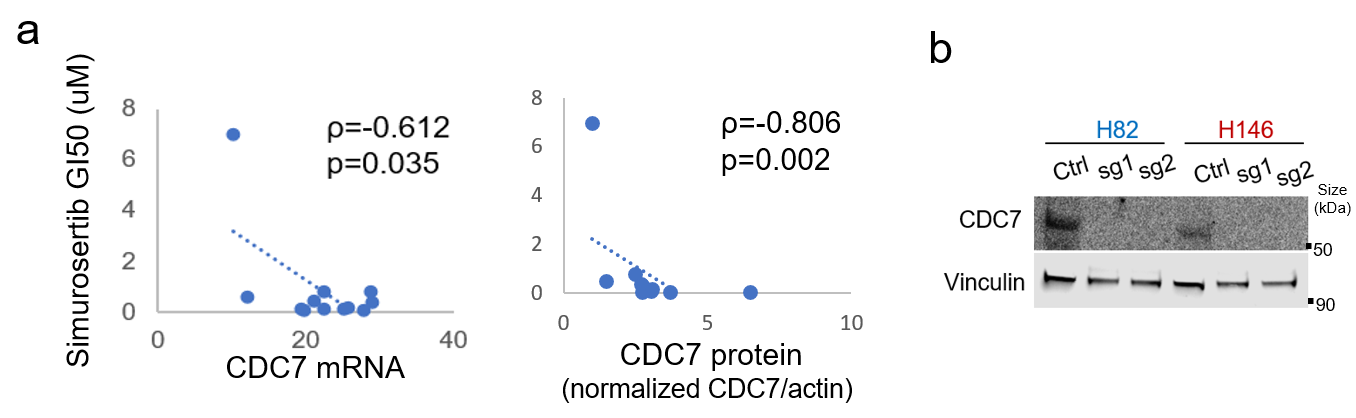

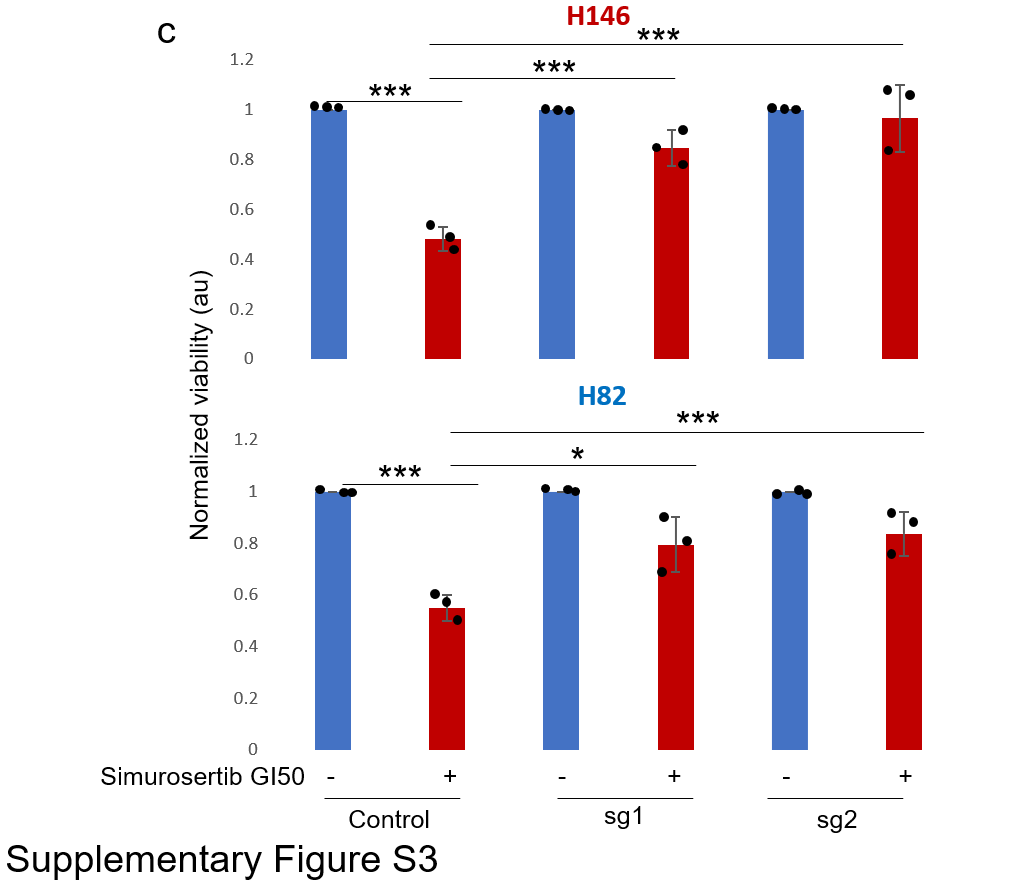


**Figure S3. Specificity of simurosertib.** (a) Correlation (Pearson) of the GI50 for simurosertib and CDC7 mRNA (left) or protein (right) levels in a panel of SCLC cell lines. (b) Western blot showing CDC7 CRISPR-Cas9 KO with two different sgRNAs in H82 (SCLC-N) and H146 (SCLC-A) cell lines. (c) Barplots showing viability after 4 day treatment with simurosertib GI50 dose in control and CDC7-KO isogenic H82 and H146 cell lines. p-values for (c) were calculated using the Student’s t-test (unpaired, heterogeneous variances, two-tailed). p-value legend: *<0.05, **<0.01, ***<0.001.

**
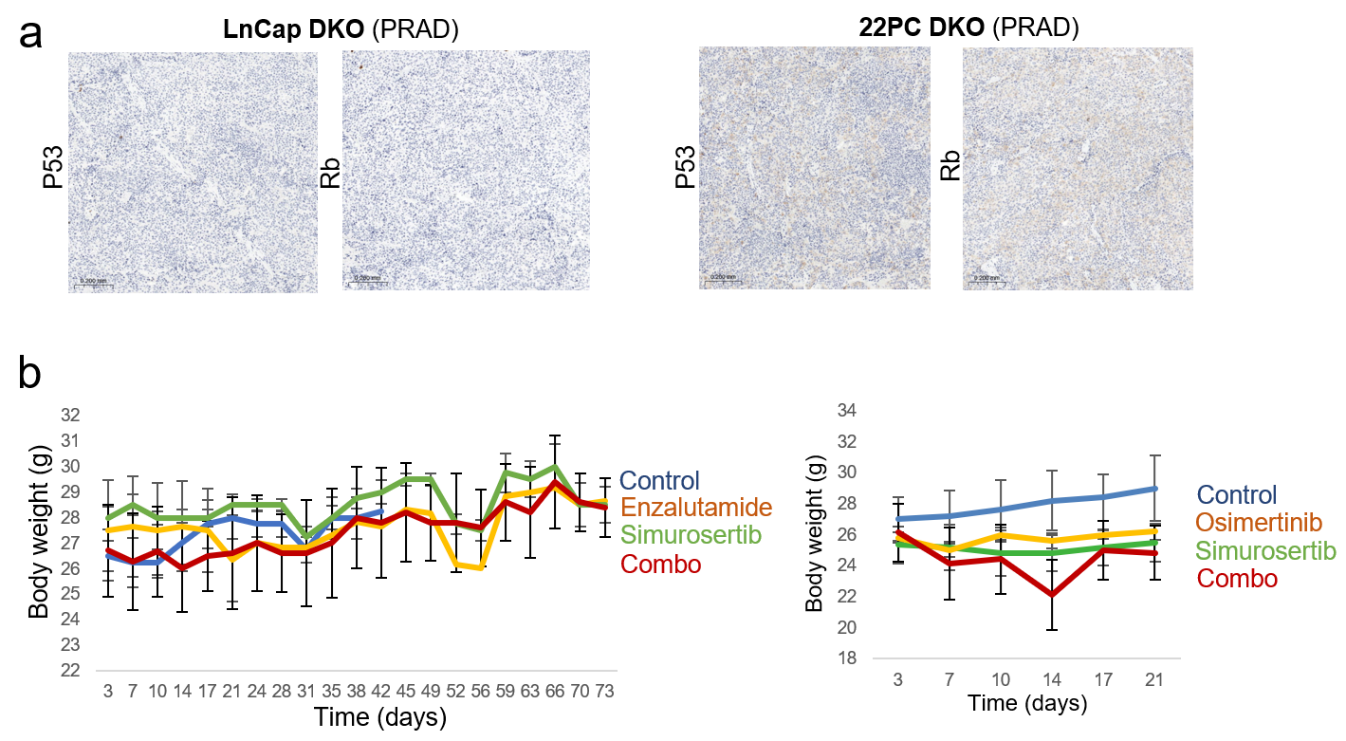
**

**
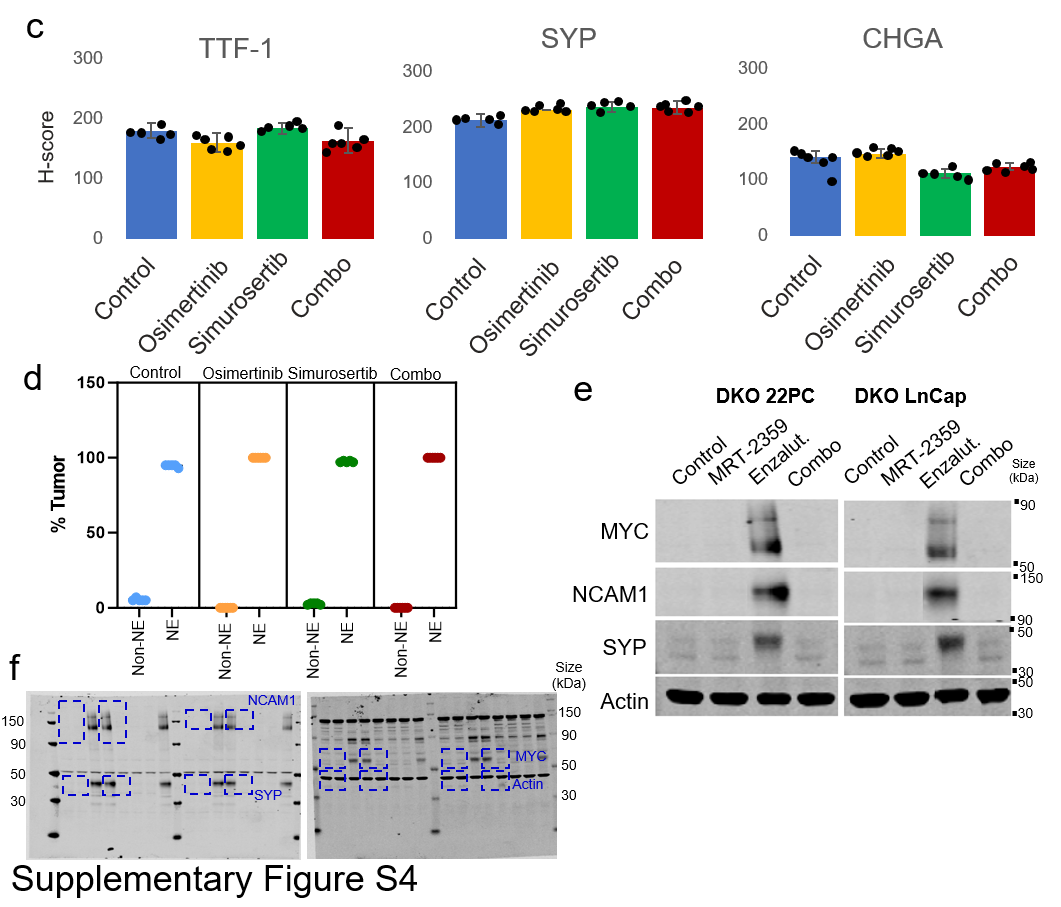
**

**Figure S4. The combination of simurosertib and targeted therapy shows limited toxicity.** (a) Representative IHC images for P53 and Rb protein expression in the PRAD models of transformation. (b) Body weight for mice treated with enzalutamide, simurosertib or their combination in experiments with TP53/RB1-inactivated LnCap/AR and 22PC, shown in Figure 3A (left); or with osimertinib, simurosertib or their combination in the experiment with Lx151 shown in Figure 3**G** (right). (c) H-score for the IHC staining for the LUAD marker TTF-1 and the NE markers SYP and CHGA in the tumors, as well as (d) percentage of NE and non-NE portions of the tumors from the Lx151 experiment depicted as mean and SD, in Figure 3**G**, collected at experimental arm endpoint. (e) Representative western blot showing MYC downregulation by MRT-2359 treatment leading to loss of the NE transformation phenotype image occurring after enzalutamide treatment. Cells were treated with enzalutamide as indicated in Figure 5d and/or 20 nM MRT-2359 for 7 days before protein collection. Image was cropped and reassembled to avoid showing irrelevant lanes. Uncropped gels are shown in (f) p-values for (b) and (c) were calculated using the Student’s t-test (unpaired, heterogeneous variances, two-tailed).


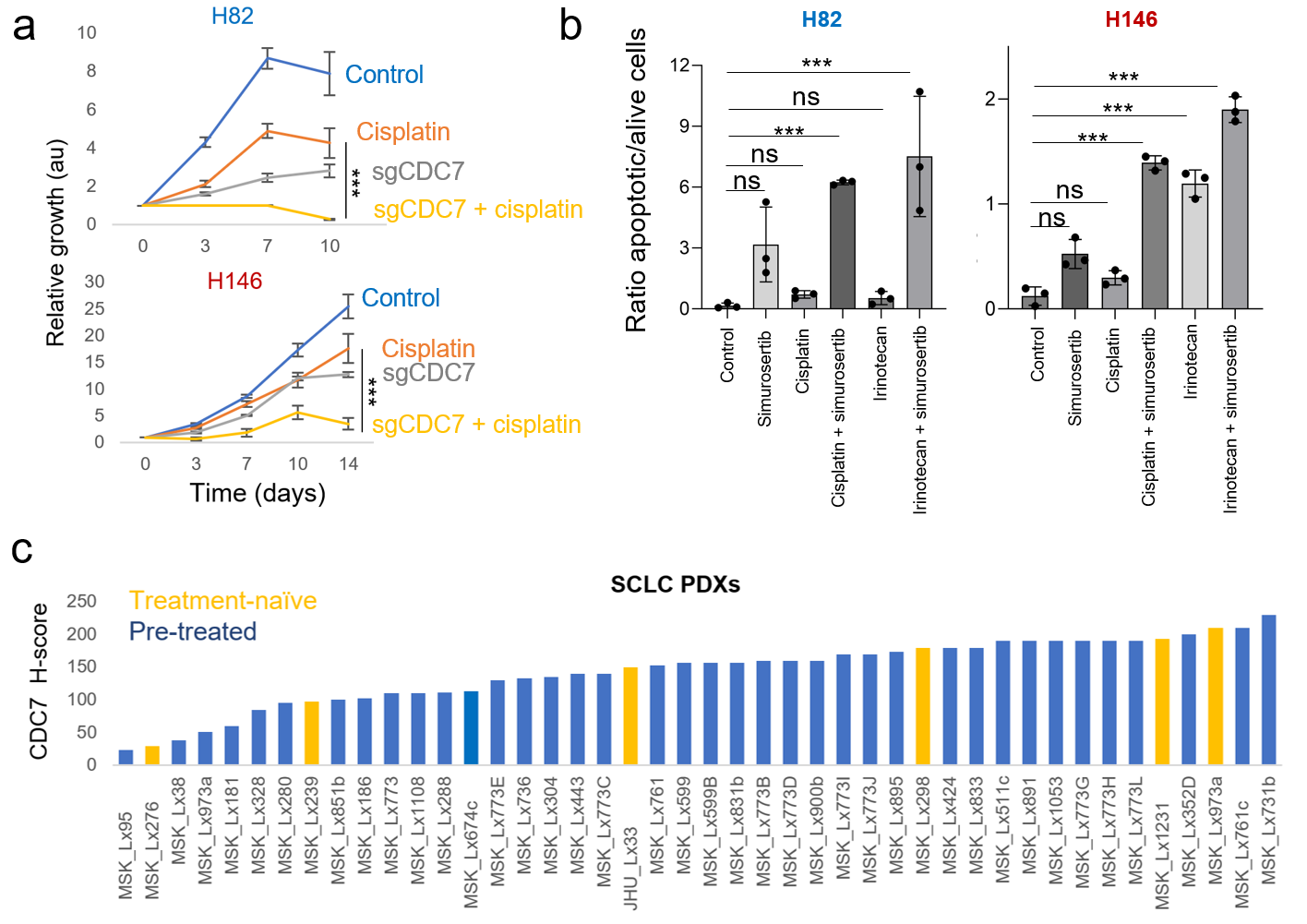

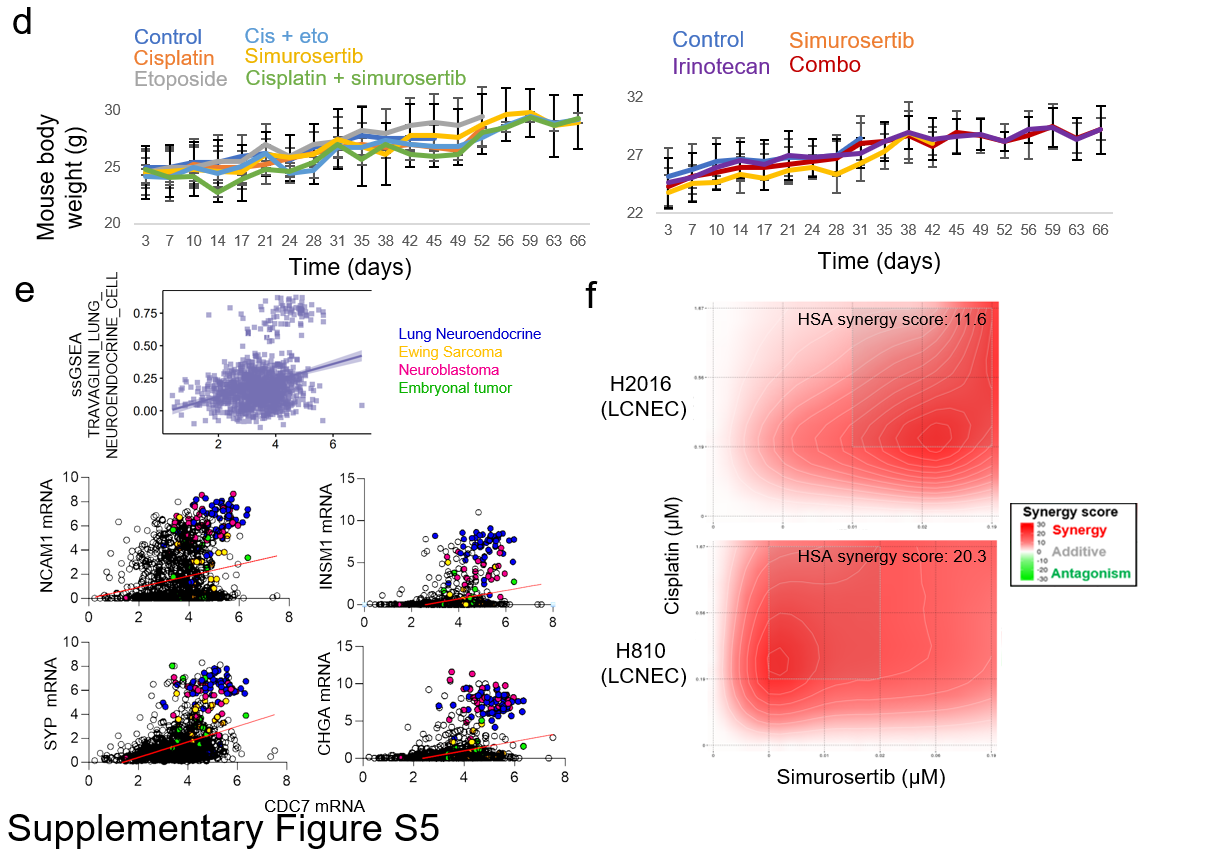


**Figure S5. Simurosertib induces sensitivity to chemotherapy.** (a) Growth curves of isogenic control and CDC7 CRISPR-Cas9 KO isogenic cell lines for H82 (SCLC-N) and H146 (SCLC-A), treated with cisplatin. Proliferation is shown as relative growth after normalization by day 0. (b) Apoptosis assays in H82 and H146 cell lines treated with cisplatin, irinotecan, simurosertib, or the combinations of cisplatin+simurosertib or irinotecan+simurosertib, shown as the ratio of apoptotic cells normalized by alive cell number. (c) IHC determination of CDC7 protein expression in our platform of SCLC PDX. H-score for CDC7 is shown, colored by the treatment status of the tumor from which each PDX was derived (treatment-naïve versus progressed on chemotherapy). (d) Body weight measurements for mice treated with cisplatin, etoposide, simurosertib, or the combinations of cisplatin+etoposide or cisplatin+simurosertib (left), or with irinotecan, simurosertib or their combination (right). (e) Correlation of CDC7 mRNA expression with enrichment (single sample GSEA, or ssGSEA) for the TRAVAGLINI_LUNG_NEUROENDOCRINE_CELL NE gene signature (top) or with NE markers (bottom). CDC7 expression is shown as logRPKM (top) or log(TPM+1) (bottom). (f) Synergy plots of the combination of cisplatin and simurosertib in LCNEC cell lines.


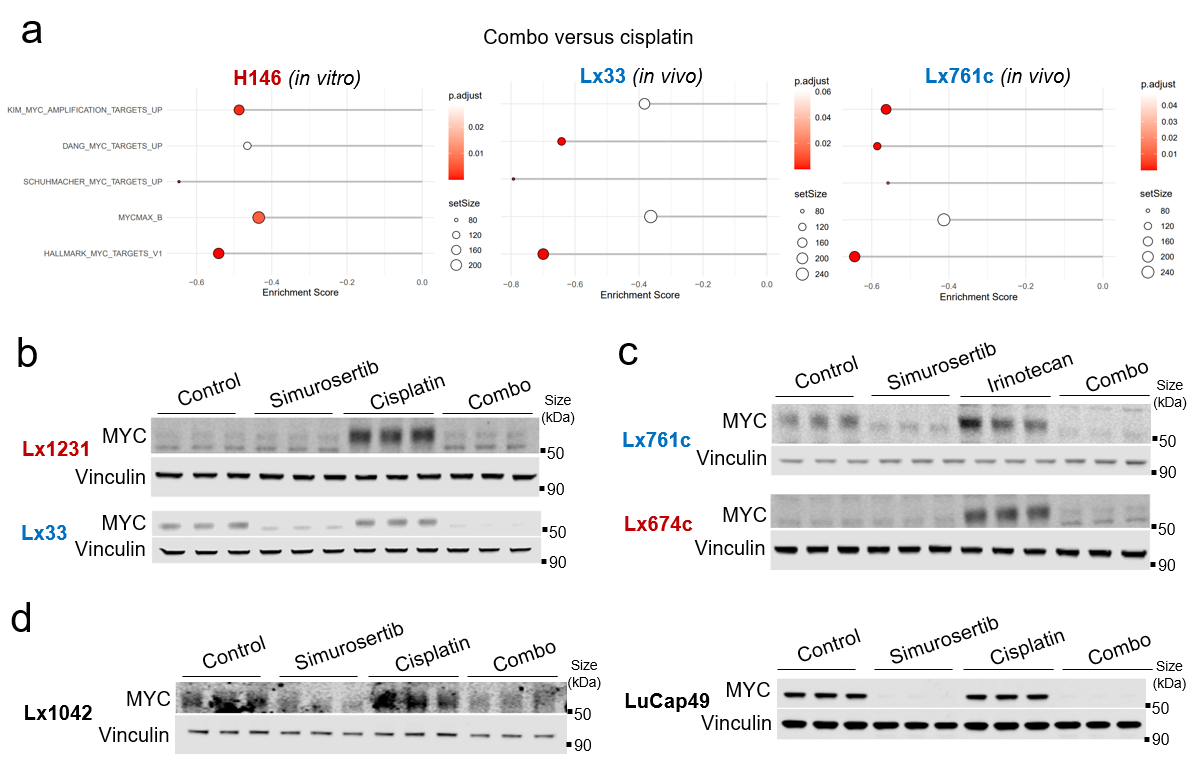


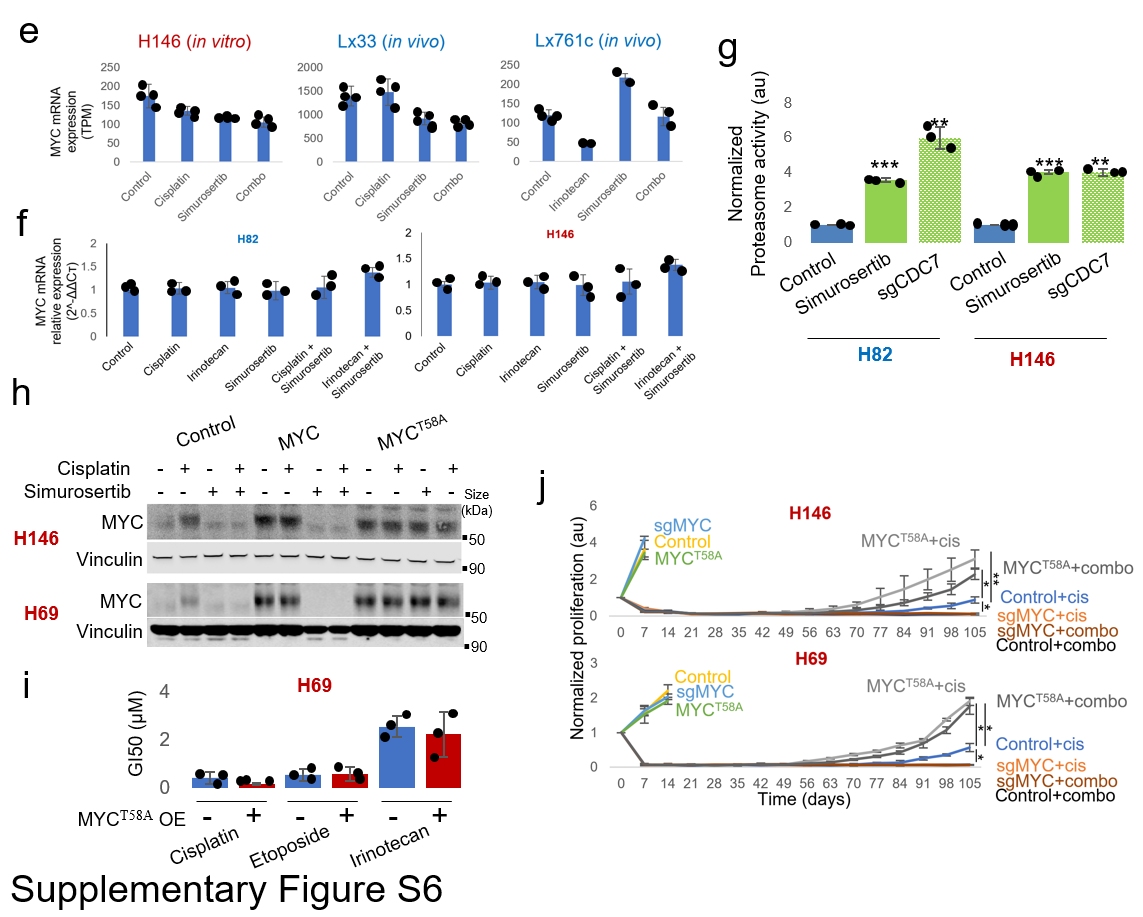


**FIgure S6. Mechanism of chemotherapy sensitization by simurosertib.** (a) Dotplots showing downregulation of genes involved in MYC pathways from a pathway enrichment analysis on DEGs from combo- versus chemotherapy in SCLC preclinical models treated *in vitro* or *in vivo* (cisplatin for H146 (SCLC-A, *in vitro*, 7 day treatment) and Lx33 (SCLC-N, *in vivo*, tumors collected at treatment arm endpoint), or irinotecan for Lx761c (SCLC-N, in vivo, tumors collected at treatment arm endpoint)). Western blot images showing MYC protein expression in treatment-naïve PDX treated with cisplatin, simurosertib or their combination (b) or chemotherapy-progressed PDX treated with irinotecan, simurosertib or their combination (c), or T-SCLC and NEPC PDX with cisplatin, simurosertib or their combination (d). (e) Barplots showing MYC mRNA expression in TPM units, as assessed by RNAseq in SCLC preclinical models treated *in vitro* or *in vivo* (cisplatin for H146 (SCLC-A, *in vitro,* 7 day treatment) and Lx33 (SCLC-N, *in vivo,* tumors collected at treatment arm endpoint), or irinotecan for Lx761c (SCLC-N, *in vivo,* tumors collected at treatment arm endpoint)). (f) Barplots showing MYC mRNA expression normalized by expression of an endogenous control, as assessed by RT-qPCR in H82 and H146 cell lines treated with cisplatin, irinotecan, simurosertib, or the combinations of cisplatin+simurosertib or irinotecan+simurosertib. (g) Barplots showing proteasome 20s activity in H82 (SCLC-N) and H146 (SCLC-A) cell lines with pharmacological (simurosertib) or genetic (CRISPR-Cas9 KO) CDC7 inhibition. Proteasome activity is shown as normalized by the control condition. (h) Western blot images showing MYC expression in isogenic cell lines derived from H146 and H69 (SCLC-A), including control, MYC^T58A^-overexpressing and MYC KO conditions for each cell line. Cell lines were treated with cisplatin, simurosertib or their combination for 7 days. Then, protein extraction and blotting was performed. (i) Barplot showing the drug concentration inhibiting 50% of growth (GI50) for cisplatin, etoposide and irinotecan (4 day treatment) in H82 and H146 cell lines. (j) Proliferation curves for cell lines described in (h), treated with cisplatin or the combination of cisplatin and simurosertib. Proliferation along time is shown was monitored by manual cell counting of technical replicates, and plotted as normalized by day 0 cell count. (i) Proliferation curves for cell lines described in (h), treated with cisplatin or the combination of cisplatin and simurosertib. Proliferation along time is shown was monitored by manual cell counting of technical replicates, and plotted as normalized by day 0 cell count. p-values were calculated using the Student’s t-test (unpaired, heterogeneous variances, two-tailed). p-value legend: *<0.05, **<0.01, ***<0.001.

**Supplementary Materials and Methods**

*Cell lines*

H1563 (CRL-5875), H660 (CRL-5813), H69 (HTB-119), H82 (HTB-175), H146 (HTB-173), DMS-114 (CRL-2066), H810 (CRL-5816) and H2106 (CRL-5923) were purchased from ATCC. LnCap and 22PC cell lines were maintained as previously described^4^. Cell lines were authenticated through the STR characterization method and regularly tested for Mycoplasma (Universal Mycoplasma Detection Kit, #30-1012K, ATCC). All experiments were performed in low passage cells. All cell lines were cultured according to ATCC guidelines or as previously described^4^.

For the generation of the Lx151 PDX-derived cell line, established PDX tumors were resected and dissociated to a single-cell suspension as previously described^5^. Briefly, tissue dissociation was performed with a gentleMACS tissue dissociator and a human tumor dissociation kit (Miltenyi). Red blood cells were lysed with ACK lysing buffer (Lonza). Cell suspensions were resuspended in RPMI1640 supplemented with 10% FBS and seeded in cell culture flasks until generation of a mixed suspension/adherent cell line.

*Plasmid vectors, virus production and transductions*

Lentiviral particles were produced and used to infect isogenic cell lines of interest, as described previously^6^, through concurrent transfection of HEK293T cells (ATCC, # CRL-1573) with a 3:2:1 ratio of lentiviral plasmid:psPAX2:pMD2.G with JetPrime transfection reagent (Polyplus, # 114-15) at a 2:1 JetPrime:DNA ratio. Medium was changed 24 h after transfection and viral supernatants were collected 72 h after transfection. Viral supernatants were syringe-filtered with a 0.45-μM PVDF filter (Millipore, # SLHVM33RS) and concentrated approximately 20-fold with Lenti-X Concentrator (Takara Bio, # 631232) according to the manufacturer’s protocol.

To generate Cas9-expressing cell lines, cells were spin-transduced with lentiviral particles made out of a lentiviral plasmid designed to constitutively express Cas9 (#125592, Addgene) as described in ^5^, and selected with blasticidin 2.5 μg/mL.

Cells were similarly spin-transduced as described in ^5^ with lentiviral particles made out of lentiviral LV03 vectors expressing sgRNAs for *CDC7* (#HSPD0000047627 and HSPD0000047628, Sigma) or the respective control vector expressing a safe targeting sgRNA BFP (#HSCONTROL_AAVS1 on LV03, Sigma), or the Lv151 vector overexpressing *CDC7* (#EX-M0793-Lv151 Genecopoeia).

*TP53/RB1*-deficient PRAD cell lines were generated as previously described^4^. *TP53/RB1*-deficient LUAD cell lines were generated by lentiviral transduction of a construct expressing a dominant negative TP53 isoform and a short hairpin RNA against *RB1*, produced from the FU-CYW vector that was previously described^7^ and kindly shared by Dr. Owen Witte.

*CRISPR-Cas9 screening*

Cells were transduced at low MOI (∼30% transduction efficiency) with our previously described druggable genome library^8^, and transduced cells were selected with puromycin (0.5 μg/mL), as described in ^6^. In this instance we used library pools 1-3, containing sgRNAs targeting genes with pharmacologic inhibitors available, either approved for clinical use, or in advanced stages of clinical development. After antibiotic selection, cell pellets were collected as day 0 time point for the screen, and cells were split into three flasks (biological replicates) with sufficient cells per flask to maintain a minimum of 1,000x library representation. The screen, gDNA extraction, and NGS PCR were carried out as previously described ^6^. Briefly, cell pellets were lysed, and genomic DNA was extracted (Qiagen) and quantified by Qubit (ThermoScientific). A quantity of gDNA covering 1000X representation of sgRNAs was PCR amplified to add Illumina adapters and multiplexing barcodes. Amplicons were quantified by Qubit and Bioanalyzer (Agilent) and sequenced on Illumina HiSeq 2500. Sequencing reads were aligned to the screened library and counts were obtained for each gRNA. FASTQ pre-processing was performed using FastX Toolkit to generate a counts file. Filtering was applied to exclude sgRNAs and samples with low counts. Guides which did not have greater than 5 counts per million were excluded. A minimum of 2 counts was required across all experimental samples, and a count per million greater than 5 in two or more samples was required. Enriched genes were identified using the Bioconductor package edgeR_3.42.4.

*Antibodies*

Antibodies for CDC7 (#3603, Cell Signaling Technology), synaptophysin (#36406, Cell Signaling Technology), CD56 (#99746, Cell Signaling Technology), AR (#5153, Cell Signaling Technology), vinculin (#13901, Cell Signaling Technology), tubulin (#3873, Cell Signaling Technology) and actin (#3700, Cell Signaling Technology). Quantifications were performed with the Image Studio software (Version 3.1, Li-Cor). Antibodies used for IHC included CDC7 (#MA5-12589 from ThermoFisher Scientific), AR (Dako #M3562) synaptophysin (Dako #A0010), and chromogranin A (Dako #A0430). IHC was performed on FFPE tissue from resected tumor samples or PDX obtained from patients with *de novo* and transforming LUAD, SCLC, PRAD and NEPC. For immunohistochemical staining, slides were deparaffinized and steamed for 45 min in Target Retrieval Solution (Dako). Immunocomplexes were detected using PV Poly-HRP anti-mouse IgG (Leica Microsystems, #PV6114) followed by a TSA biotin amplification step (Perkin Elmer) with DAB as the chromogen. Tissue sections were counterstained with hematoxylin, and slides were digitized on a Ventana DP 200 Slide Scanner (Roche). Expression was scored in a blinded manner by pathologists, whereby the optical density level (“0” for no brown color, “1” for faint and fine brown chromogen deposition “2” for intermediate chromogen deposition and “3” for prominent chromogen deposition) was multiplied by the percentage of cells at each staining level, resulting in a total H-score range of 0–300. All study subjects had provided signed informed consent for biospecimen analyses under Institutional Review Board-approved protocols (#14-209 and #14-091).

In vivo *treatments*

4-10 female (22PC, PDX) or male (LnCap/AR) 6-week-old NOD.Cg-Prkdc<scid> Il2rg<tm1Wjl>/SzJ (NSG) mice (PDX) or female 6-week-old athymic nude mice (cell line xenografts) were subcutaneously engrafted per treatment arm and until tumors reached 100-150 mm^3^. At that point, mice were randomized into groups and treated with either vehicle, cisplatin (2 mg/kg i.p. once/week), etoposide (3 mg/kg i.p. QDx3), simurosertib (40 mg/kg p.o. QDx3), irinotecan (5 mg/kg i.p. once/week), enzalutamide (10 mg/kg p.o. QDx5), osimertinib (25 mg/kg p.o. QDx5) or the combinations of cisplatin + etoposide, cisplatin + simurosertib, irinotecan + simurosertib, enzalutamide + simurosertib or osimertinib + simurosertib at the previously mentioned doses. Mice weights and tumor volumes were measured twice a week and mice were sacrificed when tumors reached humane endpoint (volume = 1000 mm^3^). The number of mice per treatment arm were selected according to previous experience with the models and response to treatments. Blinding was not performed. All animal experiments were approved by the Memorial Sloan Kettering Cancer Center (MSKCC) Animal Care and Use Committee (#13-07-007).

*Data from Cancer Cell Line Encyclopedia*

CDC7 mRNA expression data from Cancer Cell Line Encyclopedia (CCLE)^9^ was downloaded from UCSC Xenabrowser portal (<https://xenabrowser.net/>) in December 2020.

*RNAseq alignment and quantification*

Transcript abundances were quantified using RNA-seq reads by Salmon v1.1.0^10^ Raw reads of RNA-seq were mapped to 25 mer indexed hg38 genome. In addition to default settings, mapping validation (--validatemappings), bootstrapping with 30 re-samplings (--numBootstraps), sequence specific biases correction (--seqBias), coverage biases correction (--posBias) and GC biases correction (--gcBias) were also enabled. Transcripts were mapped to genes based on Ensembl 92^11^, normalized by size factor at gene level. Subsequently the differential gene expression were evaluated on Salmon output files using Sleuth v0.30.0^12^ in gene mode. Wald test was performed on differential gene expressions. Genes were marked as significantly differentially expressed if the False Discovery Rates, q, calculated using the Benjamini-Hochberg method, was less than 0.05, and beta (Sleuth-based estimation of log2 fold change) > 0.58, which approximately equivalent to a log2 fold change of 1.5.

*Publicly available RNAseq datasets analyses*

Public datasets leveraged in the present manuscript accessible through cbioportal.com include Abida et al., PNAS 2019^13^, LUAD TCGA PanCancer^14^, LUAD OncoSG, Nat Gen 2020^15^ and PRAD SU2C/PCF Dream Team, PNAS2019^13^. The public datasets were divided into four groups according to their mutation status of TP53 and RB1, as TP53WT/RB1WT, TP53MT/RB1WT, TP53WT/RB1MT, and TP53MT/RB1MT. RNAseq expression distribution of CDC7 were presented in box plots for the above four groups of samples. RNAseq expression values were downloaded through cBioPortal. <*data type 1:RSEM*> The expression quantification for LUAD ^16^ are in RSEM (RNAseq by Expectation-Maximization) that have been normalized using DESeq2 v.1.16.1 followed by log transformation whereas that for PRAD (SU2C/PCF Dream Team)^3^ are in batch normalized RSEM then followed by log transformation. <*data type 2: RSEM z-score*> Log-transformed mRNA expression z-scores compared to the expression distribution of all samples were downloaded for both LUAD and PRAD. The pairwise comparisons of mean expressions were conducted among the four groups and evaluated by Wilcoxon test. (Using traditional RNAseq DEG approach to evaluate DE p value by limma pipeline: We applied linear modelling on the normalized and log transformed RSEM values which are assumed to be normally distributed using limma (v3.28.14)^17^. The coefficients and standard errors were then estimated for each pair of contrast from the linear model. Empirical Bayes Statistics for differential expressions were carried out to evaluate the significance value).

The expression values of CDC7 were correlated in scatter plots for previously mention seven cohorts. RNAseq expression values were downloaded through cBioPortal. <*data type 1:RSEM*> The expression values are in RSEM (RNAseq by Expectation-Maximization) that have been using DESeq2 v.1.16.1 normalization, LUSD (OncoSG, Nat Genet 2020)^2^, or batch normalized followed by log transformation. <*data type 2: RSEM z-score*> Log-transformed mRNA expression z-scores compared to the expression distribution of all samples were downloaded.

*Pathway enrichment analyses*

Gene set enrichment analysis (GSEA)^18^ was conducted on the full sets of differential gene expression output from the previously mentioned comparisons. Genes were ranked by p value scores computed as -log10(p value)*(sign of beta). The annotations of gene set were taken from Molecular Signatures Database (MSigDB v7.0.1)^18,19^ of gene set enrichment was evaluated using permutation test and the p value was adjusted by Benjamini-Hochberg procedure. Any enriched gene sets with adjusted p value ≤ 0.1 were regarded as significant. This analysis was conducted using ClusterProfiler R package v3.18.1^20^. Some enriched gene sets of interests were selected, and their pathway annotations were concatenated manually to remove redundancy and achieve high generality. When the pathway terms were merged, median enrichment score was taken as the new group enrichment score, p values were aggregated using Fisher’s method from the Aggregation R package^21^, and core enrichment of genes were collapsed. The consolidated gene set enrichments were then presented in dot plots.

*ATAC-seq*

ATAC-seq dataset is available at Gene Expression Omnibus (GEO), GSE237071. The reads were trimmed for both quality and Illumina adaptor sequences using trim_galore v0.4.4 (<https://github.com/FelixKrueger/TrimGalore>) in the pair-end mode. Then the raw reads were aligned to human assembly hg38 using bowtie2 v2.3.4^22^ using the default parameters. Aligned reads with the same start site and orientation were removed using the Picard tool (<https://broadinstitute.github.io/picard/>). Enriched regions in individual samples were called using MACS2^23^ and then filtered against genomic ‘blacklisted’ regions (http://mitra.stanford.edu/kundaje/akundaje/release/blacklists/hg38-human/hg38.blacklist.bed.gz). The filtered peaks within 500 bp were merged to create a union of peak atlas. Raw read counts were tabulated over this peak atlas using featureCounts v1.6.0^24^. The read counts were then normalized with DESeq2. The read density profile in the format of bigwig file for each sample was created using the BEDTools suite (<https://bedtools.readthedocs.io>) with the normalization factor from DESeq2^25^. All bigwig genome tracks on XPO1 gene region were generated using pyGenomeTracks v3.5^26^.

*RT-qPCR*

Retrotranscription was performed with the Superscript IV VILO kit (Fisher Scientific, #11756050) following manufacturer’s instructions. The following TaqMan (ThermoFisher) probes were used: *MYC* (Hs00153408_m1) and UBC (Hs00824723_m1). Data was analyzed as previously described^27^.

*Single-cell sequencing*

PDX tumors were dissociated and processed as previously described^28,29^, leveraging a H2KD-PE antibody (Biolegend, #114718).

*Single-cell transcriptomic analyses*

The FASTQ files for the DKO LnCap/AR tumors were initially processed with CellRanger V7, using default settings and the combined human+mouse genome and transcript reference (refdata-gex-GRCh38-and-mm10-2020-A) provided by 10X Genomics. Subsequently, the output from CellRanger was analyzed using *Seurat* R package version 4. The Seurat *read10X* command was used to read the filtered barcode/feature matrix, and the percentage of mitochondrial reads per cell was calculated for subsequent filtering. Cells were excluded based on the following criteria: more than 20% mitochondrial reads, fewer than 750 genes, or less than 1,000 distinct molecules. Cells meeting any of these conditions were removed from downstream analysis. It's important to note that before applying these filters, mouse genes were excluded from the expression matrix, ensuring the effective removal of mouse cells. Finally, we assessed the cell cycle phase of each cell using Seurat's *scoreCellCycle* functions. The filtered data was normalized and scaled using Seurat's *SCTransform* method, with regression against the previously computed cell cycle scores. Post-normalization, we computed PCA coordinates, retaining the first 20 for clustering and projection analysis. Clustering was performed using Seurat's *FindNeighbors* and *FindClusters* functions, with several resolution values tested. After manual inspection, we settled on a resolution value of 0.2 for subsequent analysis. The UMAP projection was generated using *RunUMAP* with the top 20 PCA coordinates. Cluster-specific marker genes were identified using *FindAllMarkers*, with a log fold change cutoff of 0.25 and a minimum presence in 25% of cells. Finally, we calculated module scores for the following gene sets: HALLMARK_MYC_TARGETS_V1, HALLMARK_MYC_TARGETS_V2, Adeno, Transition, and NEPC. Trajectory analysis was doing using *Monocle3* (version 1.3.1). We used the R package *SeuratWrappers* to convert the Seurat objects for use in Monocle preserving the original pca mapping and UMAP reduction. The data was re-clustered with Monocle's cluster_cells function and then the trajectory graph and cell pseudotimes were computed.

The complete set of Rscripts used is available at (https://github.com/soccin/scRNA/tree/proj/p14880b)

*Visualization of scRNA-seq data*

For the PtRP GEMM dataset, to visualize cell layouts, we used either uniform manifold approximation and projection (UMAP)^30^ or force–directed layout (FDL). UMAPs were generated by creating a partition–based graph abstraction (PAGA) based on Phenograph^31^ clusters that served to initialize the low dimensional embedding. The effective minimum distance between embedded points was set to 0.2 to 0.3 (knn=30, min_dist=0.2–0.3, and init_pos=‘paga’). To visualize cell state transitions and local relationships, FDLs were constructed. We first construct an affinity graph A that follows the procedure used in MAGIC^32^. We first consider the k-nearest neighbors (kNN = 30) graph based on Euclidean distance in PCA space using 100 PCs (default). We then create affinity graph A by applying an adaptive Gaussian kernel (kernel width σ = 10) to the kNN graph that accounts for differential densities of cells in the phenotypic manifold, followed by symmetrizing the matrix. Based on A, we calculate the FDL using the draw graph function in scanpy^33^. To visualize gene expression projected on cell layouts, we normalized the UMI counts by library size, scaled by median library size, and log2-transformed with a pseudocount of 1. We chose to perform log transformation to be consistent across our other scRNA-seq study in normal mouse prostate^34^, facilitating mapping and reproducibility across datasets. To visualize normalized gene expression and distribution of NEPC and PRAD cell types at each time point, the dataset was segmented according to time point and plotted using the draw graph function in scanpy. The FDL was colored by cell type (4B) or normalized CDC7 expression (4C). The dotplot function in scanpy was used to generate dotplots in order to represent mean normalized gene expression at 8, 9, 12, and 16 weeks.

*Pseudotime Analysis*

For the PtRP GEMM dataset, pseudotime analysis using Palantir^35^was performed to model the transition of adenocarcinoma to other lineages. We first created a subset of mutant Gfp+ PtRP cells (N=16,831). Normalized counts of the top 3000 highly variable genes (excluding cell cycle genes) were projected onto the top 89 principal components selected by knee-point to explain 62% of variance. We then used Palantir^35^ to identify potential trajectories of transdifferentiation based on the inference of pseudotime. Pseudotime models transitions between cells and relies on the assumption that cells (possibly captured within the same timepoint) can be ordered along a latent dimension measuring global cell state changes over time, based on similarity in gene expression between cells. Pseudotime has been successfully applied in the context of healthy cell differentiation. However, it presumes unidirectionality in the inferred trajectories, an assumption that is less applicable in the cancer setting where bi-directional cell- state transitions can occur. Thus, caution must be taken when interpreting pseudotime in the cancer context. A caveat is that only lineage tracing can definitively demonstrate a cell state transition. Because this GEMM does not permit such lineage tracing, we instead rely on pseudotime to suggest putative trajectories of cell state transitions. Moreover, certain properties of pseudotime and the associated cell fate probabilities can be leveraged to support the existence of a putative cell state transition.

To perform pseudotime analysis, we first constructed a diffusion map (knn = 30) and retained the first 15 diffusion components based on eigengap. We determined that a reasonable start cell of the putative transition would be an adenocarcinoma cell with low Cdkn2a expression (typically overexpressed following Trp53 and Rb1 loss). Consistent with this assumption, putative terminal NEPC and Pou2f3–Gfp states display the highest Cdkn2a expression, whereas wildtype epithelial cells display no Cdkn2a expression at all. To identify an appropriate Cdkn2a–low cell, we identified the Phenograph cluster of tumor cells with the lowest imputed median Cdkn2a and randomly picked a cell within this cluster (start cell barcode: ‘PRP_9weeks_Intact_R1_157613955672309’). Palantir calculated a pseudotemporal ordering from this start cell to four ‘terminal’ states (Pou2f3–Gfp, NEPC, Tff3–Gfp, and EMT–Gfp). We focused on the transition to the NEPC ‘terminal’ state for downstream analysis.

For the PtRP GEMM dataset, to visualize MYC pathway expression in our dataset, we used gene sets MYC_TARGETS_V1 and MYC_TARGETS_V2 collected in the MSigDB Hallmark dataset (1). Given the sparse nature of single-cell sequencing that arises from gene dropout, for this analyis, we imputed gene expression using MAGIC (k= 30, t=3)^32^. First, cells were ordered according to increasing pseudotime. For each gene set, the mean MAGIC imputed gene expression of all genes was calculated. A Gaussian filter (sigma = 500) was applied in order to smooth expression for easier visualization. The matplotlib library in python was used to plot line plots of smoothened expression values. To visualize pseudotime-ordered expression of individual genes of interest, a similar procedure as above was used. Gaussian-smoothened MAGIC imputed gene expression for each gene of interest was plotted using the heatmap function from the Seaborn library in python.

REFERENCES

1. Ghandi, M. *et al.* Next-generation characterization of the Cancer Cell Line Encyclopedia. *Nature* **569**, 503–508 (2019).

2. Chen, J. *et al.* Genomic landscape of lung adenocarcinoma in East Asians. *Nat Genet* **52**, 177–186 (2020).

3. Robinson, D. *et al.* Integrative clinical genomics of advanced prostate cancer. *Cell* **161**, 1215–1228 (2015).

4. Mu, P. *et al.* SOX2 promotes lineage plasticity and antiandrogen resistance in TP53-and RB1-deficient prostate cancer. *Science (1979)* **355**, 84–88 (2017).

5. Hulton, C. H. *et al.* Direct genome editing of patient-derived xenografts using CRISPR-Cas9 enables rapid in vivo functional genomics. *Nat Cancer* **1**, 359–369 (2020).

6. Quintanal-Villalong, A. *et al.* Inhibition of XPO1 Sensitizes Small Cell Lung Cancer to First- and Second-Line Chemotherapy. *Cancer Res* **82**, 472–483 (2022).

7. Park, J. W. *et al.* Reprogramming normal human epithelial tissues to a common, lethal neuroendocrine cancer lineage. *Science (1979)* **362**, 91–95 (2018).

8. Wohlhieter, C. A. *et al.* Concurrent Mutations in STK11 and KEAP1 Promote Ferroptosis Protection and SCD1 Dependence in Lung Cancer. *Cell Rep* **33**, (2020).

9. Barretina, J. *et al.* The Cancer Cell Line Encyclopedia enables predictive modelling of anticancer drug sensitivity. *Nature* **483**, 603–607 (2012).

10. Patro, R., Duggal, G., Love, M. I., Irizarry, R. A. & Kingsford, C. Salmon provides fast and bias-aware quantification of transcript expression. *Nat Methods* **14**, 417–419 (2017).

11. Zerbino, D. R. *et al.* Ensembl 2018. *Nucleic Acids Res* **46**, D754–D761 (2018).

12. Pimentel, H., Bray, N. L., Puente, S., Melsted, P. & Pachter, L. Differential analysis of RNA-seq incorporating quantification uncertainty. *Nat Methods* **14**, 687–690 (2017).

13. Abida, W. *et al.* Genomic correlates of clinical outcome in advanced prostate cancer. *Proc Natl Acad Sci U S A* **166**, 11428–11436 (2019).

14. Weinstein, J. N. *et al.* The cancer genome atlas pan-cancer analysis project. *Nat Genet* **45**, 1113–1120 (2013).

15. Chen, J. *et al.* Genomic landscape of lung adenocarcinoma in East Asians. *Nat Genet* **52**, 177–186 (2020).

16. Chen, J. *et al.* Genomic landscape of lung adenocarcinoma in East Asians. *Nat Genet* **52**, 177–186 (2020).

17. Ritchie, M. E. *et al.* Limma powers differential expression analyses for RNA-sequencing and microarray studies. *Nucleic Acids Res* **43**, e47 (2015).

18. Subramanian, A. *et al.* Gene set enrichment analysis: A knowledge-based approach for interpreting genome-wide expression profiles. *Proc Natl Acad Sci U S A* **102**, 15545–15550 (2005).

19. Liberzon, A. *et al.* Molecular signatures database (MSigDB) 3.0. *Bioinformatics* **27**, 1739–1740 (2011).

20. Yu, G., Wang, L. G., Han, Y. & He, Q. Y. ClusterProfiler: An R package for comparing biological themes among gene clusters. *OMICS* **16**, 284–287 (2012).

21. Yi, L., Pimentel, H., Bray, N. L. & Pachter, L. Gene-level differential analysis at transcript-level resolution. *Genome Biol* **19**, 1–11 (2018).

22. Langmead, B. & Salzberg, S. L. Fast gapped-read alignment with Bowtie 2. *Nat Methods* **9**, 357–359 (2012).

23. Zhang, Y. *et al.* Model-based analysis of ChIP-Seq (MACS). *Genome Biol* **9**, (2008).

24. Liao, Y., Smyth, G. K. & Shi, W. FeatureCounts: An efficient general purpose program for assigning sequence reads to genomic features. *Bioinformatics* **30**, 923–930 (2014).

25. Love, M. I., Huber, W. & Anders, S. Moderated estimation of fold change and dispersion for RNA-seq data with DESeq2. *Genome Biol* **15**, 1–21 (2014).

26. Lopez-Delisle, L. *et al.* pyGenomeTracks: reproducible plots for multivariate genomic datasets. *Bioinformatics* **37**, 422–423 (2021).

27. Quintanal-Villalonga, Á. *et al.* FGFR1 and FGFR4 oncogenicity depends on n-cadherin and their co-expression may predict FGFR-targeted therapy efficacy. *EBioMedicine* **53**, 1–15 (2020).

28. Quintanal-Villalonga, Á. *et al.* Protocol to dissociate, process, and analyze the human lung tissue using single-cell RNA-seq. *STAR Protoc* **3**, (2022).

29. Chan, J. M. *et al.* Signatures of plasticity, metastasis, and immunosuppression in an atlas of human small cell lung cancer. *Cancer Cell* **39**, 1479-1496.e18 (2021).

30. McInnes, L., Healy, J., Saul, N. & Großberger, L. UMAP: Uniform Manifold Approximation and Projection. *J Open Source Softw* **3**, 861 (2018).

31. Levine, J. H. *et al.* Data-Driven Phenotypic Dissection of AML Reveals Progenitor-like Cells that Correlate with Prognosis. *Cell* **162**, 184–197 (2015).

32. Dijk, D. Van *et al.* Recovering Gene Interactions from Single-Cell Data Resource Recovering Gene Interactions from Single-Cell Data Using Data Diffusion. *Cell* **174**, 716-729.e27 (2018).

33. Wolf, F. A., Angerer, P. & Theis, F. J. SCANPY: Large-scale single-cell gene expression data analysis. *Genome Biol* **19**, (2018).

34. Karthaus, W. R. *et al.* *Regenerative Potential of Prostate Luminal Cells Revealed by Single-Cell Analysis*. https://www.science.org.

35. Setty, M. *et al.* Characterization of cell fate probabilities in single-cell data with Palantir. *Nat Biotechnol* **37**, 451–460 (2019).
